# Robotic Techniques in Esophagogastric Cancer Surgery: An Assessment of Short- and Long-Term Clinical Outcomes

**DOI:** 10.1245/s10434-021-11082-y

**Published:** 2021-12-10

**Authors:** Sivesh K. Kamarajah, Ewen A. Griffiths, Alexander W. Phillips, Jelle Ruurda, Richard van Hillegersberg, Wayne L. Hofstetter, Sheraz R. Markar

**Affiliations:** 1 Department of Upper Gastrointestinal Surgery, Queen Elizabeth Hospital Birmingham, University Hospitals Birmingham NHS Trust, Birmingham, UK; 2 Institute of Cancer and Genomic Sciences, College of Medical and Dental Sciences, University of Birmingham, Birmingham, UK; 3 Northern Oesophagogastric Unit, Royal Victoria Infirmary, Newcastle University Trust Hospitals, Newcastle Upon Tyne, UK; 4 School of Medical Education, Newcastle University, Newcastle Upon Tyne, Tyne and Wear UK; 5 University Medical Center Utrecht, University Utrecht, Utrecht, The Netherlands; 6 Thoracic and Cardiovascular Surgery, The University of Texas MD Anderson Cancer Center, Houston, TX USA; 7 Department of Surgery and Cancer, Imperial College London, London, UK; 8 Department of Molecular Medicine and Surgery, Karolinska Institutet, Stockholm, Sweden

## Abstract

**Background:**

Robotic esophagogastric cancer surgery is gaining widespread adoption. This population-based cohort study aimed to compare rates of textbook outcomes (TOs) and survival from robotic minimally invasive techniques for esophagogastric cancer.

**Methods:**

Data from the United States National Cancer Database (NCDB) (2010–2017) were used to identify patients with non-metastatic esophageal or gastric cancer receiving open surgery (to the esophagus, *n* = 11,442; stomach, *n* = 22,183), laparoscopic surgery (to the esophagus [LAMIE], *n* = 4827; stomach [LAMIG], *n* = 6359), or robotic surgery (to the esophagus [RAMIE], *n* = 1657; stomach [RAMIG], *n* = 1718). The study defined TOs as 15 or more lymph nodes examined, margin-negative resections, hospital stay less than 21 days, no 30-day readmissions, and no 90-day mortalities. Multivariable logistic regression and Cox analyses were used to account for treatment selection bias.

**Results:**

Patients receiving robotic surgery were more commonly treated in high-volume academic centers with advanced clinical T and N stage disease. From 2010 to 2017, TO rates increased for esophageal and gastric cancer treated via all surgical techniques. Compared with open surgery, significantly higher TO rates were associated with RAMIE (odds ratio [OR], 1.41; 95% confidence interval [CI], 1.27–1.58) and RAMIG (OR 1.30; 95% CI 1.17–1.45). For esophagectomy, long-term survival was associated with both TO (hazard ratio [HR 0.64, 95% CI 0.60–0.67) and RAMIE (HR 0.92; 95% CI 0.84–1.00). For gastrectomy, long-term survival was associated with TO (HR 0.58; 95% CI 0.56–0.60) and both LAMIG (HR 0.89; 95% CI 0.85–0.94) and RAMIG (HR 0.88; 95% CI 0.81–0.96). Subset analysis in high-volume centers confirmed similar findings.

**Conclusion:**

Despite potentially adverse learning curve effects and more advanced tumor stages captured during the study period, both RAMIE and RAMIG performed in mostly high-volume centers were associated with improved TO and long-term survival. Therefore, consideration for wider adoption but a well-designed phase 3 randomized controlled trial (RCT) is required for a full evaluation of the benefits conferred by robotic techniques for esophageal and gastric cancers.

**Supplementary Information:**

The online version contains supplementary material available at 10.1245/s10434-021-11082-y.

Esophagectomy and gastrectomy remain the mainstay of curative therapy for esophageal and gastric cancers, but surgical approach techniques vary. A growing evidence base for minimally invasive techniques appears to suggest either improved or similar morbidity without a compromise of oncologic quality.^1–3^ However, most of the studies preclude analysis comparing robotic esophagectomy or gastrectomy, which in more recent years have been gaining increasing adoption internationally.

Robotic techniques have specific advantages including a three-dimensional view and increased degrees of freedom at the wrist, which may lend to better technical surgical performance and thus better clinical outcomes for esophagectomy and gastrectomy. To date, only one single-center European randomized controlled trial (RCT)^4^ has shown improvements in postoperative complications, pain, short-term quality of life, and functional recovery with robotic versus open esophagectomy. Furthermore, a recent publication from the Upper Gastrointestinal International Robotic-Assisted Association (UGIRA) highlighted promising results from this technique when undertaken in high-volume specialized centers with adequate training.^5^

The data concerning robotic gastrectomy is based largely on observational cohort studies^6,7^ and originates from the Far East, with a different patient population and standard of lymphadenectomy from what is commonly observed in Western centers.

The quality assessment of surgical care has moved toward the use of textbook outcomes (TOs), a composite measure first developed in 2010 by colorectal surgeons in Netherlands as a more reliable global metric for assessing health care quality than individual outcome parameters.^1,8,9^ Although data on TOs have been reported across a variety of complex surgeries such as hepatectomy, pancreatectomy, and abdominal aneurysm repair.^1,2,8–10^ international data for esophagogastric surgery by surgical approach is more limited.^11–13^

The current population-based cohort study aimed to assess the short- and long-term clinical outcomes associated with the use of robotic minimally invasive techniques for the treatment of esophageal and gastric cancers in the United States.

## Methods

### Data Source

The National Cancer Database (NCDB) is a joint project of the Commission on Cancer (CoC) of the American College of Surgeons and the American Cancer Society.^14,15^ The NCDB gathers information from approximately 1500 CoC-accredited hospitals and includes more than 70% of all newly diagnosed malignancies in the United States of America (USA). It contains specific details about patient demographics (age, sex, race, insurance status), facility type and location, tumor characteristics (size, grade, stage, histology), treatment course (type of surgery, receipt of chemotherapy, and radiation therapy), and outcomes (resection margins, lymph node status, hospital length of stay, short- and long-term mortality).

### Study Population

#### Inclusion Criteria

The current study enrolled any patients with a diagnosis of non-metastatic esophageal (i.e., adenocarcinoma, squamous cell carcinoma) or gastric adenocarcinoma (Supplementary Table S1)) according to the International Classification of Disease for Oncology, third edition (ICD-O-3) who received esophagectomy or gastrectomy between 2010 and 2016 in the de-identified NCDB.

#### Exclusion Criteria

The exclusion criteria ruled out other histology subtypes such as mucinous tumors, neuroendocrine tumors, and other histologies; patients who underwent endoscopic resection; other concurrent cancer diagnoses; and patients with metastatic esophageal or gastric cancer.

### Study Definitions

The following patient-level characteristics as provided by NCDB were analyzed: age (18–35, 36–50, 51–65, 66– 80, ≥ 81 years), race (white, other), Charlson/Deyo comorbidity score,^16^ year of diagnosis, insurance status (Medicare, Medicaid, private insurance, uninsured), zip code-level education status (< 7%, 7–12.9%, 13–20.9%, ≥ 21%), zip code-level median household income (< $48,000, $48,000–62,999, ≥ $63,000), and urban versus rural area of residence. The zip code level education status represents the proportion of adults in the patient's zip code who did not graduate from high school categorized as equally proportioned quartiles among all U.S. zip codes.

The following hospital-level characteristics also were analyzed: center volume (quintiles 1–5), facility type (academic, community, other), facility location (Midwest, Northeast, South, West), and hospital distance from the patient’s residence (< 12.5, 12.5–49.9, ≥ 50.0 miles). Center volume was defined according to the annual surgical volume at each individual hospital derived from the unique center identification number. These then were split into five equal groups for center volume.

Finally, the study analyzed the following clinicopathologic characteristics: clinical T status (T1, T2, T3, T4, Tx) and N status (N0, N1, N2, N3, Nx), receipt of neoadjuvant therapy, tumor grade/differentiation (well/moderate, poor/anaplastic), margin status (positive, negative), and lymphovascular invasion (absent, present, unknown). Neoadjuvant therapy was defined as none, neoadjuvant chemotherapy, or neoadjuvant chemoradiotherapy. In both the group receiving neoadjuvant chemotherapy and the group receiving chemoradiotherapy, only patients receiving multi-agent chemotherapy were considered.

### Outcome Measures

The primary outcome was the rate of TOs, with TO defined as margin-negative resections, 15 or more lymph nodes examined, no prolonged hospital stay (≥21 days), no 90-day postoperative mortality, and no readmission 30 days or less after discharge. A TO was achieved when all these parameters were fulfilled. The definition used for TO in this study was derived from the original study by Busweiler et al.^17^ The secondary outcome was long-term survival, defined as the time from surgery to the last known follow-up visit or death.

### Statistical Analysis

Categorical variables were compared using the chi-square test. Non-normally distributed data were analyzed using the Mann-Whitney *U* test. Survival was estimated using Kaplan-Meier survival curves and compared using the log-rank test. A multilevel logistic regression model was used to produce an adjusted odds ratio (OR) and a 95% confidence interval (95% CI) to determine the association between surgical approach and TO. Multivariable analyses used Cox proportional hazards models.

In all models, patient-level, hospital-level, and tumor-level characteristics were included. Importantly, year of diagnosis was included to adjust for changes in developments in patient selection, diagnostic staging, multimodality treatment, and perioperative care (e.g., prehabilitation). Subset analyses were performed in high-volume centers to assess the impact of open, laparoscopic, and robotic techniques on TO and survival for both esophagectomy and gastrectomy patients. Post hoc analyses also were performed to compare outcomes for laparoscopic and robotic techniques.

A *p* value lower than 0.05 was considered statistically significant. Data analysis was performed using R Foundation Statistical software (R 3.2.2) with TableOne, ggplot2, Hmisc, Matchit and survival packages (R Foundation for Statistical Computing, Vienna, Austria), as previously reported.^18^

## Results

### Esophageal Cancer

#### Baseline Characteristics

This study identified 17,947 patients with esophageal cancer treated with esophagectomy, of which 27% (*n* = 4827) were a conventional total or hybrid minimally invasive esophagectomy (LAMIE) and 9% (*n* = 1,657) were a robot-assisted minimally invasive esophagectomy (RAMIE). The patients receiving RAMIE were more commonly treated in high-volume centers (27% vs 24% vs 23%; *p* < 0.001) and academic centers (67% vs 65% vs 59%; *p* < 0.001) compared with LAMIE and open surgery. Furthermore, the patients receiving RAMIE were more likely older patients with advanced T and N stage than those treated with conventional LAMIE or open surgery. Baseline demographics are presented in Table 1.

**Table 1:** Baseline clinicopathologic characteristics of patients undergoing esophagectomy for esophageal cancer by surgical approach

	Open (*n* = 11,463) *n* (%)	Laparoscopic (*n* = 4827) *n* (%)	Robotic (*n* = 1657) *n* (%)	*p* value
*Center volume*				
1 (Lowest)	1422 (12.4)	400 (8.3)	128 (7.7)	< 0.001
2	2062 (18.0)	758 (15.7)	309 (18.6)	
3	2632 (23.0)	1056 (21.9)	376 (22.7)	
4	2749 (24.0)	1433 (29.7)	396 (23.9)	
5 (Highest)	2584 (22.6)	1177 (24.4)	450 (27.1)	
*Facility type*				
Community	3225 (28.1)	1076 (22.3)	302 (18.2)	< 0.001
Integrated	1474 (12.9)	612 (12.7)	251 (15.1)	
Academic	6758 (59.0)	3143 (65.1)	1106 (66.7)	
*Facility location*				
Northeast	2277 (19.9)	1477 (30.6)	369 (22.2)	< 0.001
Midwest	3704 (32.3)	1116 (23.1)	404 (24.4)	
South	3943 (34.4)	1352 (28.0)	669 (40.3)	
West	1533 (13.4)	886 (18.3)	217 (13.1)	
*Hospital distance (from patient’s residence) miles*				
<12.5	4789 (41.8)	1995 (41.3)	704 (42.4)	0.7
12.5–49.9	3729 (32.5)	1615 (33.4)	551 (33.2)	
≥50 miles	2939 (25.7)	1221 (25.3)	404 (24.4)	
*Year of diagnosis*				
2010–2011	3291 (28.7)	934 (19.3)	162 (9.8)	< 0.001
2012–2013	3032 (26.5)	1100 (22.8)	354 (21.3)	
2014–2015	1414 (12.3)	605 (12.5)	260 (15.7)	
2016–2017	3720 (32.5)	2192 (45.4)	883 (53.2)	
*Age at diagnosis (years)*				
18–35	76 (0.7)	27 (0.6)	10 (0.6)	0.036
36–50	967 (8.5)	378 (7.8)	124 (7.5)	
51–65	5555 (48.6)	2279 (47.3)	746 (45.0)	
66–80	4585 (40.1)	2024 (42.0)	736 (44.4)	
80+	256 (2.2)	114 (2.4)	42 (2.5)	
*Sex*				
Male	9397 (82.0)	3985 (82.5)	1378 (83.1)	0.5
Female	2060 (18.0)	846 (17.5)	281 (16.9)	
*Race*				
White	10501 (91.7)	4485 (92.8)	1517 (91.4)	0.029
Other	956 (8.3)	346 (7.2)	142 (8.6)	
*CDCC score*				
0	7950 (69.4)	3311 (68.5)	1167 (70.3)	0.3
1	2609 (22.8)	1116 (23.1)	373 (22.5)	
2	634 (5.5)	288 (6.0)	73 (4.4)	
3+	264 (2.3)	116 (2.4)	46 (2.8)	
*Insurance status*				
Medicare	5099 (45.4)	2182 (46.0)	818 (50.2)	< 0.001
Medicaid	695 (6.2)	306 (6.5)	83 (5.1)	
Private	4927 (43.9)	2102 (44.4)	691 (42.4)	
Not insured/other	507 (4.5)	149 (3.1)	36 (2.2)	
*Education level (%)*				
>21	2584 (22.6)	1146 (23.7)	408 (24.6)	0.007
13–20.9	2750 (24.0)	1033 (21.4)	382 (23.0)	
7–12.9	3612 (31.5)	1530 (31.7)	520 (31.3)	
<7	2511 (21.9)	1122 (23.2)	349 (21.0)	
*Medical income ($)*				
≤47,999	4154 (36.3)	1577 (32.6)	550 (33.2)	< 0.001
48,000–62,999	2927 (25.5)	1201 (24.9)	403 (24.3)	
63,000+	4376 (38.2)	2053 (42.5)	706 (42.6)	
*Residence*				
Metro	8656 (75.6)	3786 (78.4)	1362 (82.1)	< 0.001
Urban	1967 (17.2)	737 (15.3)	182 (11.0)	
Rural	834 (7.3)	308 (6.4)	115 (6.9)	
*AJCC clinical T stage*				
cT1	1920 (16.8)	947 (19.6)	244 (14.7)	< 0.001
cT2	2041 (17.8)	913 (18.9)	304 (18.3)	
cT3	5699 (49.7)	2338 (48.4)	917 (55.3)	
cT4	253 (2.2)	80 (1.7)	29 (1.7)	
cTx	1544 (13.5)	553 (11.4)	165 (9.9)	
*AJCC clinical N stage*				
cN0	5405 (47.2)	2336 (48.4)	798 (48.1)	< 0.001
cN1	4021 (35.1)	1678 (34.7)	605 (36.5)	
cN2	1110 (9.7)	494 (10.2)	174 (10.5)	
cN3	208 (1.8)	84 (1.7)	23 (1.4)	
cNx	713 (6.2)	239 (4.9)	59 (3.6)	
*Histology*				
Adenocarcinoma	9255 (80.8)	3967 (82.1)	1367 (82.4)	0.065
SCC	2202 (19.2)	864 (17.9)	292 (17.6)	
*Neoadjuvant therapy*				
None	3313 (28.9)	1470 (30.4)	373 (22.5)	< 0.001
NCRT	6888 (60.1)	2895 (59.9)	1116 (67.3)	
NAC	1256 (11.0)	466 (9.6)	170 (10.2)	
*Tumor grade*				
Well	727 (6.3)	402 (8.3)	115 (6.9)	< 0.001
Moderate	4519 (39.4)	1937 (40.1)	647 (39.0)	
Poor	4539 (39.6)	1822 (37.7)	639 (38.5)	
Anaplastic	1672 (14.6)	670 (13.9)	258 (15.6)	
*AJCC pathologic T stage*				
pT0	2084 (18.2)	940 (19.5)	383 (23.1)	< 0.001
pT1	3101 (27.1)	1506 (31.2)	475 (28.6)	
pT2	1671 (14.6)	713 (14.8)	254 (15.3)	
pT3	3431 (29.9)	1291 (26.7)	436 (26.3)	
pT4	147 (1.3)	37 (0.8)	12 (0.7)	
pTx	1023 (8.9)	344 (7.1)	99 (6.0)	
*AJCC pathologic N stage*				
pN0	7063 (61.6)	3108 (64.3)	1098 (66.2)	< 0.001
pN1	2129 (18.6)	866 (17.9)	323 (19.5)	
pN2	959 (8.4)	402 (8.3)	132 (8.0)	
pN3	436 (3.8)	144 (3.0)	39 (2.4)	
pNx	870 (7.6)	311 (6.4)	67 (4.0)	
*Lymphovascular invasion*				
Absent	6451 (56.3)	2842 (58.8)	949 (57.2)	0.005
Present	1945 (17.0)	747 (15.5)	243 (14.6)	
Unknown	3061 (26.7)	1242 (25.7)	467 (28.1)	
*30-Day mortality*				
No	11048 (96.4)	4723 (97.8)	1609 (97.0)	< 0.001
Yes	409 (3.6)	108 (2.2)	50 (3.0)	

*CDCC* Charlson-Deyo Score, *AJCC* American Joint Committee on Cancer, *SCC* squamous cell carcinoma, *NCRT* neoadjuvant chemoradiotherapy, *NAC* neoadjuvant chemotherapy

#### Textbook Outcome (TO)

The individual rates of TO parameters were 94% for margin-negative resections, 46% for 15 or more lymph nodes examined, 86% for a hospital stay of 21 days or less, 92% for no 30-day readmission, and 93% for no 90-day postoperative mortality. The individual rate of TO parameters was significantly higher with RAMIE than with LAMIE or open esophagectomy for margin-negative resections (96% vs 95% vs 93%; *p* < 0.001), 15 or more lymph nodes examined (55% vs 53% vs 42%; *p* < 0.001), and hospital stay of 21 days or less (88% vs 88% vs 85%; *p* < 0.001), but did not differ for 30-day readmissions (Table 2).

**Table 2:** Individual textbook parameters for patients undergoing esophagectomy for esophageal cancer or gastrectomy for gastric cancer by surgical approach

	Open *n* (%)	Laparoscopic *n* (%)	Robotic *n* (%)	*p* Value	*p* Value^a^
*Esophagectomy*					
Regional nodes examined					
< 15	6637 (57.9)	2255 (46.7)	754 (45.4)	< 0.001	0.4
≥ 15	4820 (42.1)	2576 (53.3)	905 (54.6)		
Margin status					
Negative	10697 (93.4)	4565 (94.5)	1596 (96.2)	< 0.001	0.008
Positive	760 (6.6)	266 (5.5)	63 (3.8)		
Length of stay (days)					
≤ 21	9782 (85.4)	4261 (88.2)	1461 (88.1)	< 0.001	0.9
> 21	1675 (14.6)	570 (11.8)	198 (11.9)		
90-Day mortality					
No	10582 (92.4)	4570 (94.6)	1561 (94.1)	< 0.001	0.5
Yes	875 (7.6)	261 (5.4)	98 (5.9)		
30-Day readmission					
No	10502 (91.8)	4476 (92.7)	1532 (92.5)	0.172	0.7
Yes–unplanned	154 (1.3)	51 (1.1)	15 (0.9)		
Yes–planned	786 (6.9)	300 (6.2)	110 (6.6)		
*Gastrectomy*					
Regional nodes examined					
< 15	10555 (47.5)	2671 (41.9)	646 (37.5)	< 0.001	0.001
≥ 15	11673 (52.5)	3704 (58.1)	1075 (62.5)		
Margin status					
Negative	19272 (86.7)	5839 (91.6)	1611 (93.6)	< 0.001	0.007
Positive	2956 (13.3)	536 (8.4)	110 (6.4)		
Hospital stay (days)					
≤ 21	2216 (10.0)	534 (8.4)	143 (8.3)	< 0.001	1.0
> 21	20012 (90.0)	5841 (91.6)	1578 (91.7)		
90-Day mortality					
No	20348 (91.5)	6024 (94.5)	1643 (95.5)	< 0.001	0.1
Yes	1880 (8.5)	351 (5.5)	78 (4.5)		
30-Day readmission					
No	20194 (91.0)	5894 (92.7)	1599 (93.1)	< 0.001	0.4
Yes–unplanned	398 (1.8)	75 (1.2)	14 (0.8)		
Yes–planned	1591 (7.2)	390 (6.1)	105 (6.1)		

^a^Indicates post hoc analyses comparing laparoscopic and robotic surgeries

The overall prevalence of TOs observed was 36% (*n* = 6,528) and significantly higher for the patients receiving RAMIE than for those receiving LAMIE and or open surgery (44% vs 43% vs 32%; *p* < 0.001) (Tables 2, 3). In the adjusted analysis, the patients receiving either LAMIE (OR, 1.35, 95% CI 1.26–1.46; *p* < 0.001) or RAMIE (OR, 1.41; 95% CI 1.27–1.58; *p* < 0.001) had a greater proportion of TOs than those treated with open esophagectomy (Supplementary Table S2).

**Table 3:** Textbook outcomes and long-term survival of patients undergoing esophagectomy for esophageal cancer or gastrectomy and gastric cancer by surgical approach and stratified analysis in high-volume centers

	Textbook outcomes *n* (%)	Median overall survival Months (range)	Adjusted HR (95% CI)	*p* Value	Adjusted HR (95% CI)^a^	*p* Value^a^
*All patients*						
Esophagectomy						
Open	3691 (32.3)	45.0 (43.0–46.9)	Reference			
Laparoscopic	2104 (43.6)	54.4 (50.3–60.4)	0.96 (0.91–1.01)	0.1	Reference	
Robotic	733 (44.2)	56.7 (50.9–63.0)	0.92 (0.84–1.00)	0.049	0.95 (0.87–1.05)	0.3
Gastrectomy						
Open	8624 (38.9)	42.5 (40.8–44.1)	Reference			
Laparoscopic	3034 (47.7)	63.6 (58.5–68.5)	0.89 (0.85–0.94)	< 0.001	Reference	
Robotic	884 (51.5)	66.4 (58.0–84.9)	0.88 (0.81–0.96)	0.006	0.99 (0.90–1.09)	0.8
*Subset analysis in high-volume centers*						
Esophagectomy						
Open	1072 (41.5)	51.7 (48.2–57.1)	Reference			
Laparoscopic	638 (54.3)	57.9 (48.2–66.5)	1.15 (0.98–1.35)	0.1	Reference	
Robotic	264 (58.8)	73.1 (63.0–NR)	1.72 (1.37–2.15)	< 0.001	0.89 (0.72–1.09)	0.3
Gastrectomy						
Open	1021 (62.8)	63.9 (59.0–70.0)	Reference			
Laparoscopic	2332 (52.4)	86.3 (75.3–NR)	0.95 (0.86–1.04)	0.3	Reference	
Robotic	1114 (60.2)	NR (70.7–NR)	0.82 (0.73–0.91)	0.003	0.95 (0.89–1.4)	0.7

*HR* hazard ratio, *CI* confidence interval; *NR* not reached

^a^Indicates post-hoc analyses comparing laparoscopic and robotic surgery

#### Association Between TO and Survival

**Fig. 1:**
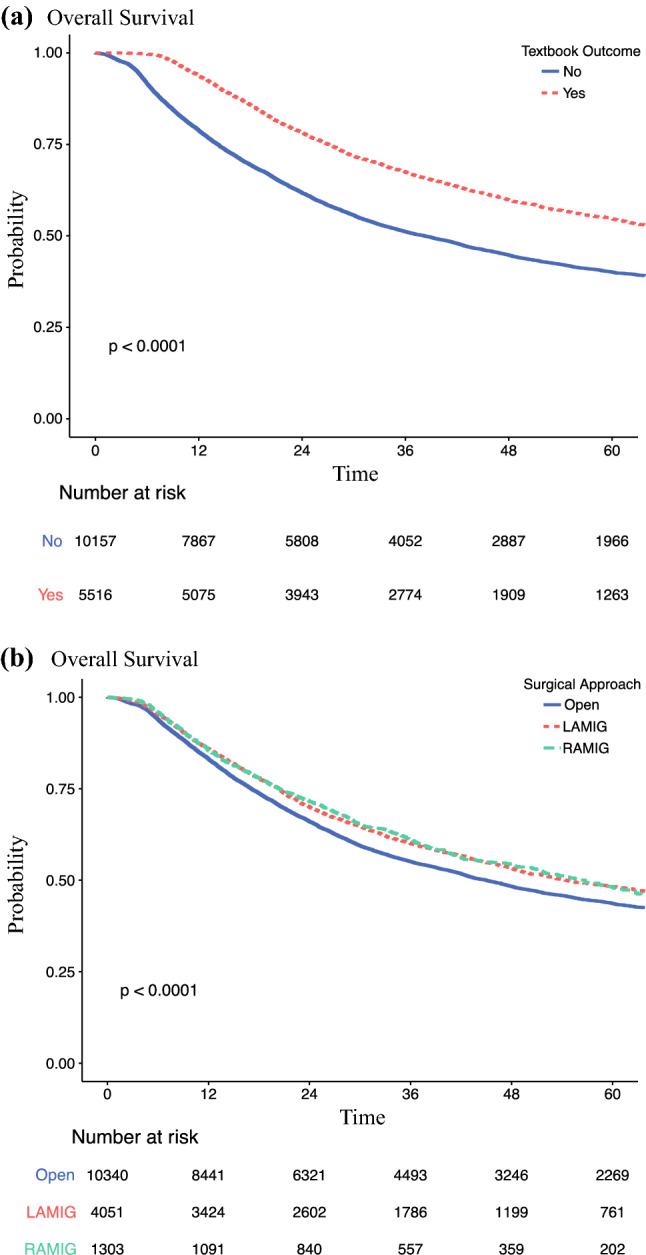
Overall survival of patients undergoing esophagectomy for esophageal cancer stratified by **A** textbook outcome and **B** surgical approach.

The patients achieving TO had a significantly longer survival than those without TO (median, 70.5 vs 38.2 months; *p* < 0.001) (Fig. 1A). The patients receiving RAMIE or LAMIE had a significantly longer survival than those treated with open esophagectomy (median, 56.7 vs 54.4 vs 45.0 months; *p* < 0.001; Fig. 1B). In the adjusted analyses, significantly improved overall survival was associated with TO (hazard ratio [HR], 0.64; 95% CI 0.60–0.67; *p* < 0.001) and RAMIE (HR 0.92; 95% CI 0.84–1.00; *p* = 0.049) (Tables 3 and Supplementary S3). However, long-term survival did not differ significantly between RAMIE and LAMIE (HR 0.92; 95% CI 0.84–1.00; *p* = 0.3; Table 3).

#### Sensitivity Analysis of TO

Sensitivity analyses were performed accounting for length of stay ≤10 days in the TO. The patients receiving RAMIE had a significantly higher TO than those treated with LAMIE or open surgery (44% vs 43% vs 32%; *p* < 0.001), which remained consistent in the adjusted analysis (Supplementary Table S2). The patients achieving TO had a significantly longer survival than those without TO (median, 71.1 vs 42.1 months; *p* < 0.001). Adjusted analyses showed that the patients receiving RAMIE had a significantly longer survival than those treated with open esophagectomy (HR 0.91; 95% CI 0.84–1.00; *p* = 0.048; Supplementary Table S4).

#### TO High-Volume (Quintile 5) Centers

Subset analyses were performed including high-volume centers (*n* = 4211), of which 28% were LAMIE and 11% were RAMIE. The baseline demographics for open surgery, LAMIE, and RAMIE are presented in Supplementary Table S5. The individual TOs are presented in Supplementary Table S6.

The individual rate of TO parameters was significantly higher with RAMIE than with LAMIE or open esophagectomy for margin-negative resections (97% vs 94% vs 95%; *p* = 0.044) and 15 or more lymph nodes examined (70% vs 67% vs 52%; *p* < 0.001), but did not differ for length of stay or 30-day readmissions (Supplementary Table S6). The overall prevalence of TOs observed was 47% (*n* = 1974) and significantly higher for the patients receiving RAMIE than for those treated with LAMIE or open surgery (59% vs 54% vs 42%; *p* < 0.001; Table 3).

In the adjusted analysis, only the patients receiving RAMIE had a greater proportion of TOs (OR, 1.72; 95% CI 1.37–2.15; *p* < 0.001) than those treated with open esophagectomy (Supplementary Table S7). The patients achieving TO had a significantly longer survival than those without TO (median, 80.4 vs 42.1 months; *p* < 0.001; Supplementary Figure S1A). The patients receiving RAMIE or LAMIE had a significantly longer survival than those treated with open esophagectomy (median, 73.1 vs 57.9 vs 51.7 months; *p* < 0.001; Supplementary Figure S1B).

In the adjusted analyses, significantly better overall survival was associated with TO (HR 0.58; 95% CI 0.52–0.64; *p* < 0.001) and RAMIE (HR 0.81; 95% CI 0.68–0.96; *p* = 0.017) (Table 3, Supplementary Table S8). However, long-term survival did not differ significantly between RAMIE and LAMIE (HR 0.99; 95% CI 0.90–1.09; *p* = 0.8) (Table 3).

### Gastric Cancer

#### Baseline Characteristics

The study identified 30,324 patients with gastric cancer, of which 21% (*n* = 6375) received laparoscopic surgery (LAMIG) and 6% (*n* = 1721) received robot-assisted minimally invasive (RAMIG). The patients receiving RAMIG were more commonly treated in high-volume centers (32% vs 29% vs 20%; *p* < 0.001) and academic centers (58% vs 57% vs 45%; *p* < 0.001), had a higher annual medical income, and had higher rates of neoadjuvant chemoradiotherapy/chemotherapy than those treated with LAMIG or open gastrectomy. Baseline demographics are presented in Table 4.

**Table 4:** Baseline clinicopathologic characteristics of patients undergoing gastrectomy for gastric cancer by surgical approach

	Open *n* (%)	Laparoscopic *n* (%)	Robotic *n* (%)	*p* Value
*Center volume*				
1 (lowest)	3425 (15.4)	686 (10.8)	143 (8.3)	< 0.001
2	4525 (20.4)	997 (15.6)	227 (13.2)	
3	4925 (22.2)	1135 (17.8)	388 (22.5)	
4	4894 (22.0)	1702 (26.7)	409 (23.8)	
5 (highest)	4459 (20.1)	1855 (29.1)	554 (32.2)	
*Facility type*				
Community	8748 (39.4)	1897 (29.8)	457 (26.6)	< 0.001
Integrated	3405 (15.3)	873 (13.7)	269 (15.6)	
Academic	10075 (45.3)	3605 (56.5)	995 (57.8)	
*Facility location*				
Northeast	4853 (21.8)	2018 (31.7)	524 (30.4)	< 0.001
Midwest	4868 (21.9)	1265 (19.8)	327 (19.0)	
South	8575 (38.6)	1786 (28.0)	552 (32.1)	
West	3932 (17.7)	1306 (20.5)	318 (18.5)	
*Hospital distance (miles)*				
< 12.5	12978 (58.4)	3517 (55.2)	904 (52.5)	< 0.001
12.5–49.9	6087 (27.4)	1829 (28.7)	542 (31.5)	
≥ 50 miles	3163 (14.2)	1029 (16.1)	275 (16.0)	
*Year of diagnosis*				
2010–2011	6507 (29.3)	1163 (18.2)	153 (8.9)	< 0.001
2012–2013	6067 (27.3)	1497 (23.5)	325 (18.9)	
2014–2015	2738 (12.3)	869 (13.6)	246 (14.3)	
2016–2017	6916 (31.1)	2846 (44.6)	997 (57.9)	
*Age at diagnosis (years)*				
18–35	240 (1.1)	75 (1.2)	19 (1.1)	< 0.001
36–50	1856 (8.4)	486 (7.6)	163 (9.5)	
51–65	7319 (33.0)	2185 (34.3)	617 (35.9)	
66–80	9769 (44.0)	2846 (44.7)	773 (45.0)	
80+	2997 (13.5)	769 (12.1)	145 (8.4)	
*Sex*				
Male	15020 (67.6)	4478 (70.2)	1258 (73.1)	< 0.001
Female	7208 (32.4)	1897 (29.8)	463 (26.9)	
*Race*				
White	16044 (72.2)	4801 (75.3)	1311 (76.2)	< 0.001
Other	6184 (27.8)	1574 (24.7)	410 (23.8)	
*CDCC score*				
0	14291 (64.3)	4161 (65.3)	1143 (66.4)	0.319
1–2	7115 (32.0)	1977 (31.0)	513 (29.8)	
2	1680 (7.6)	477 (7.5)	109 (6.3)	
3+	822 (3.7)	237 (3.7)	65 (3.8)	
*Insurance status*				
Medicare	12042 (54.8)	3368 (53.5)	920 (53.9)	< 0.001
Medicaid	1685 (7.7)	501 (8.0)	109 (6.4)	
Private	7151 (32.6)	2232 (35.5)	637 (37.3)	
Not insured/other	1077 (4.9)	190 (3.0)	40 (2.3)	
*Education level (%)*				
> 21	6634 (29.8)	1853 (29.1)	504 (29.3)	< 0.001
13%–20.9	5379 (24.2)	1392 (21.8)	372 (21.6)	
7%–12.9	6089 (27.4)	1852 (29.1)	516 (30.0)	
< 7	4126 (18.6)	1278 (20.0)	329 (19.1)	
*Medical income ($)*				
47,999	8678 (39.0)	2189 (34.3)	533 (31.0)	< 0.001
48,000–62,999	5298 (23.8)	1569 (24.6)	421 (24.5)	
63,000+	8252 (37.1)	2617 (41.1)	767 (44.6)	
*Residence*				
Metro	18612 (83.7)	5331 (83.6)	1466 (85.2)	0.493
Urban	2530 (11.4)	738 (11.6)	173 (10.1)	
Rural	1086 (4.9)	306 (4.8)	82 (4.8)	
*AJCC clinical T stage*				
cT1	3668 (16.5)	1385 (21.7)	328 (19.1)	< 0.001
cT2	2756 (12.4)	929 (14.6)	323 (18.8)	
cT3	6525 (29.4)	2097 (32.9)	613 (35.6)	
cT4	1444 (6.5)	246 (3.9)	76 (4.4)	
cTx	7835 (35.2)	1718 (26.9)	381 (22.1)	
*AJCC clinical N stage*				
cN0	11976 (53.9)	3687 (57.8)	956 (55.5)	< 0.001
cN1	4433 (19.9)	1384 (21.7)	461 (26.8)	
cN2	1608 (7.2)	445 (7.0)	134 (7.8)	
cN3	668 (3.0)	118 (1.9)	19 (1.1)	
cNx	3543 (15.9)	741 (11.6)	151 (8.8)	
*Neoadjuvant therapy*				
None	14450 (65.0)	3774 (59.2)	794 (46.1)	< 0.001
NCRT	3663 (16.5)	1449 (22.7)	555 (32.2)	
NAC	4115 (18.5)	1152 (18.1)	372 (21.6)	
*Tumor grade*				
Well	1495 (6.7)	517 (8.1)	125 (7.3)	< 0.001
Moderate	7436 (33.5)	2372 (37.2)	616 (35.8)	
Poor	11579 (52.1)	2950 (46.3)	801 (46.5)	
Anaplastic	1718 (7.7)	536 (8.4)	179 (10.4)	
*AJCC pathologic T stage*				
pT0	1085 (4.9)	453 (7.1)	186 (10.8)	< 0.001
pT1	4948 (22.3)	1967 (30.9)	520 (30.2)	
pT2	3100 (13.9)	973 (15.3)	249 (14.5)	
pT3	7604 (34.2)	2011 (31.5)	516 (30.0)	
pT4	4489 (20.2)	722 (11.3)	175 (10.2)	
pTx	1002 (4.5)	249 (3.9)	75 (4.4)	
*AJCC pathologic N stage*				
pN0	9994 (48.8)	3474 (57.2)	1001 (60.6)	< 0.001
pN1	3984 (19.5)	1093 (18.0)	303 (18.3)	
pN2	3373 (16.5)	797 (13.1)	194 (11.7)	
pN3	1864 (9.1)	347 (5.7)	76 (4.6)	
pNx	1262 (6.2)	367 (6.0)	78 (4.7)	
*Lymphovascular invasion*				
Absent	10435 (46.9)	3467 (54.4)	963 (56.0)	< 0.001
Present	8377 (37.7)	1945 (30.5)	458 (26.6)	
Unknown	3416 (15.4)	963 (15.1)	300 (17.4)	
*30-Day mortality*				
No	21259 (95.6)	6203 (97.3)	1683 (97.8)	< 0.001
Yes	969 (4.4)	172 (2.7)	38 (2.2)	

*CDCC* Charlson-Deyo Score, *AJCC* American Joint Committee on Cancer, *NCRT* neoadjuvant chemoradiotherapy, *NAC* neoadjuvant chemotherapy

#### TO

The individual rate of TO parameters was 88% for margin-negative resections, 54% for 15 or more lymph nodes examined, 91% for hospital stay of 21 days or longer, 92% for no 30-day readmission, and 92% for no 90-day postoperative mortality. The individual rates of TO parameters were significantly higher with RAMIG than with LAMIG or open gastrectomy for margin-negative resections (94% vs 92% vs 87%; *p* < 0.001),15 or more lymph nodes examined (63% vs 58% vs 53%; *p* < 0.001), and hospital stay of 21 days or longer (92% vs 92% vs 90%; *p* < 0.001) (Table 2). The overall TO rate was 41% (*n* = 12,542) and significantly higher for the patients receiving RAMIG than for those treated with LAMIG or open gastrectomy (52% vs 48% vs 39%; *p* < 0.001) (Table 3). The adjusted analysis showed a significantly increased rate of TO for the patients receiving LAMIG (OR, 1.19; 95% CI 1.12–1.26; *p* < 0.001) or RAMIG (OR, 1.30; 95% CI 1.17–1.45, *p* < 0.001) than for those treated with open gastrectomy (Supplementary Table S9).

#### Association Between TO and Survival

**Fig. 2:**
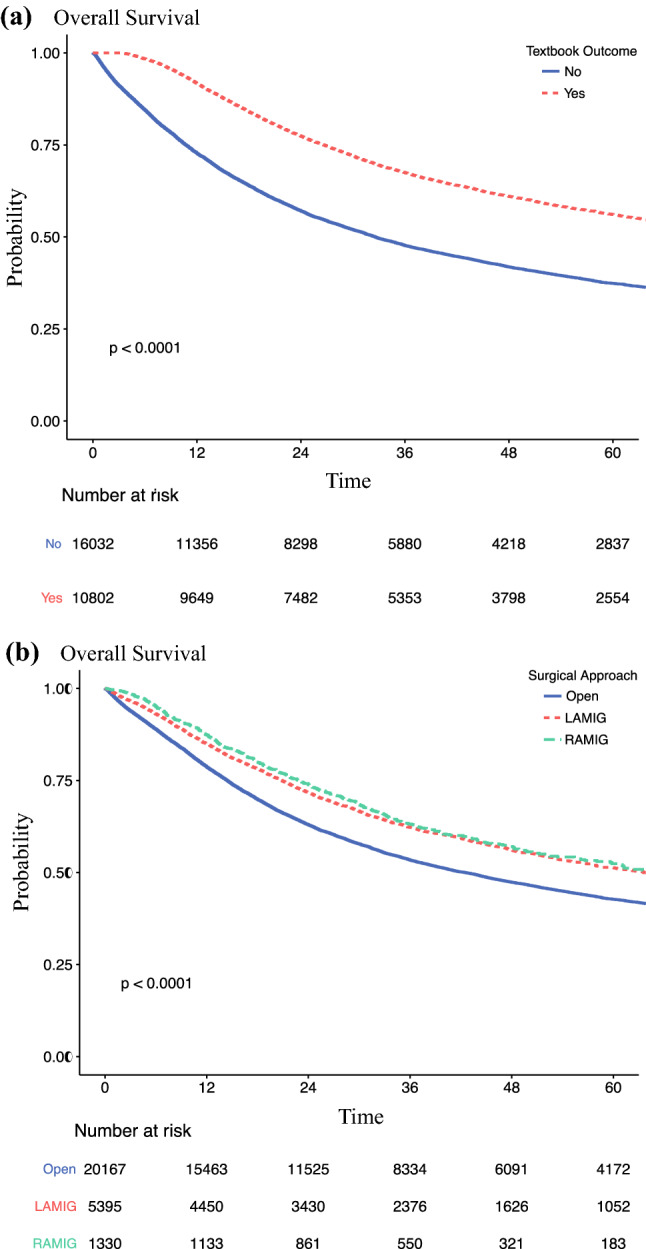
Overall survival of patients undergoing gastrectomy for gastric cancer stratified by **A** textbook outcome and **B** surgical approach.

The patients achieving TO had significantly better survival than those without TO (median, 79.6 vs 32.6 months; *p* < 0.001; Fig. 2A). The patients receiving RAMIG or LAMIG had a significantly better survival than those treated with open gastrectomy (median, 66.4 vs 63.6 vs 42.5 months; *p* < 0.001; Fig. 2B). After adjustment for potential confounding factors, a significantly longer overall survival was associated with TO (HR 0.58; 95% CI 0.56–0.60; *p* < 0.001), LAMIG (HR 0.89; 95% CI 0.85– 0.94; *p* < 0.001), and RAMIG (HR 0.88; 95% CI 0.81–0.096; *p* = 0.006) (Table 2, Supplementary Table S10). However, long-term survival did not differ significantly between RAMIG and LAMIG (HR 0.89; 95% CI 0.72–1.09; *p* = 0.3; Table 3).

#### Sensitivity Analysis of TO

Sensitivity analyses were performed accounting for a hospital stay of 10 days or longer in the TO. The patients receiving RAMIE had a significantly higher TO than those treated with LAMIE or open surgery (43% vs 39% vs 30%; *p* < 0.001; Supplementary Table S3). The patients achieving TO had a significantly longer survival than those without TO (median, 87.6 vs 35.0 months; *p* < 0.001). Adjusted analyses showed that the patients receiving RAMIE had significantly longer survival than those treated with open esophagectomy (HR 0.90; 95% CI 0.82–0.98; *p* = 0.019; Supplementary Table S3).

#### TO High-Volume (Quintile 5) Centers

Subset analyses were performed including high-volume centers (*n* = 6860), of which 27% (*n* = 1850) were LAMIG and 8% (*n* = 554) were RAMIG. Baseline demographics between open surgery, LAMIG, and RAMIG are presented in Supplementary Table S11. The individual TOs are presented in Supplementary Table S5. The individual rates of TO parameters were significantly higher with RAMIG than with LAMIG or open gastrectomy for margin-negative resections (94% vs 94% vs 92%; *p* = 0.002), 15 or more lymph nodes examined (79% vs 72% vs 65%; *p* < 0.001), and 30-day readmissions (6% vs 6% vs 9%; *p* < 0.001), but did not differ for length of stay or 30-day readmissions (Supplementary Table S5).

The similar rates of margin-negative resections between LAMIG and RAMIG may be explained by the higher rates of advanced tumors (cT3/T4) with RAMIG than with LAMIG (60.6% vs 53.7%; Supplementary Table S4). The overall prevalence of TOs observed was 56% (*n* = 3804) and was significantly higher for the patients receiving RAMIG than for those treated with LAMIG or open surgery (65% vs 60% vs 52%; *p* < 0.001; Table 3). The adjusted analysis showed a greater proportion of TOs for the patients receiving LAMIG (OR, 1.22; 95% CI 1.09–1.37; *p* = 0.001) or RAMIG (OR, 1.45; 95% CI 1.19–1.76; *p* < 0.001) than for those treated with open gastrectomy (Supplementary Table S12). The patients achieving TO had a significantly longer survival than those without TO (median, 102.9 vs 45.7 months; *p* < 0.001; Fig. 2A). The patients receiving RAMIG or LAMIG had a significantly longer survival than those treated with open esophagectomy (median, NR vs 86.3 vs 63.9 months; *p* < 0.001; Supplementary Figure S2B). In adjusted analyses, TO (HR: 0.58; 95% CI 0.54 - 0.63) <0.001) and RAMIG (HR: 0.82; 95% CI 0.73 - 0.91) 0.003) were associated with significantly improved overall survival (Table 3, Supplementary Table S13). However, long-term survival did not differ significantly between RAMIG and LAMIG (HR 0.95; 95% CI 0.89–1.04; *p* = 0.7; Table 3).

## Discussion

This national population-based cohort study demonstrated that a robot-assisted minimally invasive approach for esophageal and gastric cancers was associated with an increased rate of TOs and improved long-term survival after adjustment for potential confounding factors, and in subset analysis within high-volume centers. This study suggests that robotic surgery in centers with a sufficient case load may confer some advantages when undertaken by appropriately trained surgeons, and also suggests a rationale for wider dissemination of robotic techniques to improve outcomes from complex esophageal and gastric cancer surgeries. Although the outcomes are comparable between laparoscopic and robotic techniques, strategies to allow dissemination of robotic surgical techniques in the context of complex cancer surgery require careful thought, with credentialing, standardization training programs, audit, and performance evaluation with video analysis before independent practice is permitted.^19^

The rapid increase in adoption of robotic surgery during the past decade is attributable to a few main benefits. First, the robotic technology is thought to improve feasibility and reproducibility, likely shortening the learning curve^20^ compared with the ergonomically challenging laparoscopic approaches. Second, this platform allows for three-dimensional visualization, a magnified view, and improved ergonomics with enhanced stability and maneuverability through the use of articulated wristed instruments controlled from a remote console, with better visualization of tissue planes and deep neurovascular structures.^21^ This allows for a more precise and accurate dissection in narrow spaces, obese patients, and bulky, locally advanced tumors. Difficulties in exposure and the inherent limitations of rigid instrumentation can affect not only the dissection during laparoscopic surgery, but also the completeness of resection margins. Collectively, these advantages translate to lower conversion rates, shorter operative time, fewer postoperative intensive care unit admissions, and a shorter hospital stay.^12,22,23^ Furthermore, robotic surgery appears to be linked with increased odds of margin-negative resection and improved lymphadenectomy, suggesting that these approaches may offer oncologic advantages beyond the benefits of short-term improvements in postoperative recovery.^12^ These marginal gains translate to improvement in long-term survival, as reported in the current study.

During the next few years, dissemination of robotic surgery is key to ensuring its routine adoption into clinical practice to optimize patient benefits. First, implementation of training programs should be safe and be adopted in high-volume centers and/or surgeons to ensure a critical case load to shorten any potential proficiency gain curve. Second, regulators and the surgical community need to have systems for tightly controlled auditing, such as an international registry, in place to monitor performance of robotic surgeries across various specialties. Because accurate data are necessary to inform the creation of appropriate safeguards, national bodies should consider providing coverage for robotic surgery with provisions for evidence development.^24^ The Upper GI International Robotic Association (UGIRA) was established to facilitate the reporting of robotic procedures worldwide and to analyze variation and learning curves.^3,5,19^ Use of these provisions would facilitate greater understanding of how robotic procedures are used in real-world practice. Akin to post-market surveillance of pharmaceuticals, such provisions also would create a common data resource from which the comparative safety and effectiveness of robotic operations could be evaluated by numerous investigators. Third, video-based analyses of performance and telemedicine for surgical coaching should be used to shorten the learning curve among surgeons. Finally, additional adjuncts such as image-based surgery with projections of preoperative imaging may allow refined surgical anatomy and dissection in cancer surgery.

This study had some important limitations. First, certain inherent biases with access to hospital services offering robotic surgery may be associated with TOs such as improved oncology services, access to research trials, and improved failure to rescue. These biases may contribute to overall better outcomes. However, these data provide us impetus for future qualitative research that may help explain such differences. Second, the NCDB does not distinguish whether longitudinal (proximal and distal) or circumferential margins were involved, prohibiting ability to assess their relative importance. Third, the observed findings may relate to a cluster effect whereby patients likely to receive a robotic procedure are likely to have better performance status and be more likely to travel to academic centers.^25^ Fourth, understanding the health economic costs of the robotic platform is key. Although robotic surgery in this study was associated with higher rates of TOs through reduced overall complications and need for intervention or return to the intensive treatment unit than open or laparoscopic surgery, robotic surgery likely may have lower costs to the hospitals in the long term. However, these data are not available in the NCDB, which mandates further research. Finally, patient-related outcomes such as quality of life were not included in the TOs, which could be important because desired outcomes might differ between physicians and patients.^26^

## Conclusion

Despite potentially adverse learning curve effects and more advanced tumor stages captured during the study period, both RAMIE and RAMIG, as performed in mostly high-volume centers, were associated with improved TOs and long-term survival. Therefore, consideration for their wider adoption but also a well-designed phase 3 RCT is required for a full evaluation of the benefits conferred by robotic techniques for esophageal and gastric cancers.

## Supplementary Information

Below is the link to the electronic supplementary material.

Supplementary file1 (DTD 66 kb)
