# Supplementary material for: Robotic Techniques in Esophagogastric Cancer Surgery: An Assessment of Short- and Long-Term Clinical Outcomes
Source: Ann Surg Oncol. 2021 Dec 10;29(5):2812–25. doi: 10.1245/s10434-021-11082-y (PMC8989809; doi:10.1245/s10434-021-11082-y)
Supplement: Supplementary file 1 — Supplementary file1 (DTD 66 kb) [file 10434_2021_11082_MOESM1_ESM.docx]

**TABLE S1** Summary of codes used in National Cancer Database to derive variables included in this study

| Tumor histology |  |
| --- | --- |
| Adenocarcinoma | 8140– 8146 |
| Surgery codes |  |
| Esophagectomy/gastrectomy | 30, 40, 50–55, 80 |

**TABLE S2** Logistic regression of factors associated with textbook outcome for patients undergoing esophagectomy for esophageal cancer

|  |  | OR (univariable) *p* Value | OR (multivariable) *p* Value |
| --- | --- | --- | --- |
| Center volume | 1 (lowest) | — | — |
|  | 2 | 1.59 (1.38–1.82) <0.001 | 1.50 (1.30–1.72) <0.001 |
|  | 3 | 1.83 (1.61–2.08) <0.001 | 1.69 (1.47–1.95) <0.001 |
|  | 4 | 3.28 (2.89–3.72) <0.001 | 2.83 (2.44–3.28) <0.001 |
|  | 5 (highest) | 3.60 (3.18–4.09) <0.001 | 3.28 (2.81–3.84) <0.001 |
| Facility type | Community | — | — |
|  | Integrated | 1.39 (1.25–1.55) <0.001 | 1.00 (0.88–1.13) 0.979 |
|  | Academic | 2.02 (1.88–2.18) <0.001 | 1.06 (0.96–1.18) 0.263 |
| Facility location | Northeast | — | — |
|  | Midwest | 0.59 (0.55–0.65) <0.001 | 0.62 (0.56–0.68) <0.001 |
|  | South | 0.56 (0.51–0.60) <0.001 | 0.67 (0.61–0.73) <0.001 |
|  | West | 0.78 (0.70–0.86) <0.001 | 0.97 (0.87–1.08) 0.568 |
| Hospital distance (miles) | <12.5 | — | — |
|  | 12.5–49.9 | 1.17 (1.09–1.25) <0.001 | 1.03 (0.95–1.12) 0.417 |
|  | ≥50 | 1.40 (1.29–1.51) <0.001 | 1.13 (1.02–1.25) 0.023 |
| Year of diagnosis | 2010–2011 | — | — |
|  | 2012–2013 | 1.21 (1.11–1.33) <0.001 | 1.21 (1.10–1.33) <0.001 |
|  | 2014–2015 | 1.53 (1.38–1.71) <0.001 | 1.51 (1.35–1.69) <0.001 |
|  | 2016–2017 | 1.74 (1.61–1.89) <0.001 | 1.79 (1.64–1.95) <0.001 |
| Age at diagnosis (years) | 18–35 | — | — |
|  | 36–50 | 0.92 (0.62–1.36) 0.663 | 1.29 (0.84–2.00) 0.248 |
|  | 51–65 | 0.82 (0.56–1.20) 0.302 | 1.15 (0.76–1.77) 0.510 |
|  | 66–80 | 0.77 (0.53–1.13) 0.174 | 1.11 (0.73–1.71) 0.628 |
|  | 80+ | 0.64 (0.42–0.99) 0.041 | 0.94 (0.59–1.53) 0.811 |
| Sex | Male | — | — |
|  | Female | 0.94 (0.87–1.02) 0.128 | 0.98 (0.90–1.07) 0.711 |
| Race | White | — | — |
|  | Other | 0.81 (0.72–0.91) <0.001 | 1.00 (0.88–1.14) 0.985 |
| CDCC score | 0 | — | — |
|  | 1 | 0.95 (0.88–1.02) 0.141 | 0.97 (0.89–1.04) 0.373 |
|  | 2 | 1.02 (0.90–1.17) 0.726 | 1.01 (0.88–1.17) 0.864 |
|  | 3+ | 0.79 (0.64–0.97) 0.025 | 0.81 (0.65–1.00) 0.057 |
| Insurance status | Medicare | — | — |
|  | Medicaid | 0.84 (0.73–0.96) 0.013 | 0.87 (0.74–1.02) 0.090 |
|  | Private | 1.23 (1.15–1.31) <0.001 | 1.14 (1.05–1.25) 0.003 |
|  | Not Insured / Other | 0.74 (0.62–0.87) 0.001 | 0.77 (0.64–0.93) 0.007 |
| Education level (%) | >21 | — | — |
|  | 13–20.9 | 0.99 (0.90–1.08) 0.838 | 1.06 (0.95–1.17) 0.289 |
|  | 7–12.9 | 1.15 (1.06–1.25) 0.001 | 1.09 (0.99–1.21) 0.072 |
|  | <7 | 1.24 (1.13–1.36) <0.001 | 1.09 (0.98–1.21) 0.109 |
| Medical income ($) | ≤47,999 | — | — |
|  | 48,000–62,999 | 1.12 (1.04–1.22) 0.004 | 1.03 (0.94–1.13) 0.464 |
|  | 63,000+ | 1.32 (1.23–1.42) <0.001 | 1.13 (1.03–1.24) 0.012 |
| Residence | Metro | — | — |
|  | Urban | 0.88 (0.81–0.95) 0.002 | 0.91 (0.82–1.00) 0.057 |
|  | Rural | 1.30 (1.15–1.46) <0.001 | 1.12 (0.98–1.27) 0.089 |
| AJCC clinical T stage | cT1 | — | — |
|  | cT2 | 0.98 (0.89–1.09) 0.712 | 1.03 (0.92–1.16) 0.597 |
|  | cT3 | 1.07 (0.98–1.16) 0.136 | 1.10 (0.98–1.24) 0.093 |
|  | cT4 | 0.79 (0.62–0.99) 0.046 | 0.89 (0.69–1.15) 0.387 |
|  | cTx | 0.75 (0.67–0.85) <0.001 | 0.92 (0.80–1.06) 0.244 |
| AJCC clinical N stage | cN0 | — | — |
|  | cN1 | 1.11 (1.04–1.19) 0.002 | 1.06 (0.98–1.14) 0.178 |
|  | cN2 | 1.23 (1.11–1.36) <0.001 | 1.09 (0.97–1.22) 0.168 |
|  | cN3 | 1.27 (1.01–1.59) 0.041 | 1.11 (0.87–1.42) 0.410 |
|  | cNx | 0.74 (0.64–0.85) <0.001 | 0.97 (0.81–1.15) 0.687 |
| Histology | Adenocarcinoma | — | — |
|  | SCC | 0.77 (0.71–0.83) <0.001) | 0.82 (0.75–0.90) <0.001 |
| Neoadjuvant therapy | None | — | — |
|  | NCRT | 1.07 (1.00–1.15) 0.050 | 0.88 (0.80–0.97) 0.008 |
|  | NAC | 1.05 (0.94–1.17) 0.375 | 0.91 (0.80–1.04) 0.177 |
| Surgical approach | Open | — | — |
|  | Laparoscopic | 1.62 (1.51–1.74) <0.001 | 1.35 (1.26–1.46) <0.001 |
|  | Robotic | 1.67 (1.50–1.85) <0.001 | 1.42 (1.27–1.58) <0.001 |

OR, odds ratio; CDCC,; AJCC, American Joint Committee on Cancer; SCC, squamous cell carcinoma; NCRT, n**eoadjuvant chemoradiotherapy**; NAC, n**eoadjuvant chemotherapy**

**TABLE S3** Cox regression of factors associated with long-term survival for patients undergoing esophagectomy for esophageal cancer

|  |  | HR (univariable) *p* Value | HR (multivariable) *p* Value |
| --- | --- | --- | --- |
| Center volume | 1 (lowest) | — | — |
|  | 2 | 0.90 (0.83–0.97) 0.007 | 0.93 (0.86–1.01) 0.086 |
|  | 3 | 0.83 (0.77–0.90) <0.001 | 0.88 (0.81–0.95) 0.002 |
|  | 4 | 0.67 (0.62–0.73) <0.001 | 0.78 (0.71–0.86) <0.001 |
|  | 5 (highest) | 0.69 (0.64–0.74) <0.001 | 0.79 (0.71–0.87) <0.001 |
| Facility type | Community | — | — |
|  | Integrated | 0.84 (0.78–0.90) <0.001 | 1.00 (0.92–1.08) 0.909) |
|  | Academic | 0.76 (0.73–0.80) <0.001 | 0.96 (0.90–1.03) 0.250) |
| Facility location | Northeast | — | — |
|  | Midwest | 1.09 (1.03–1.16) 0.005 | 0.97 (0.91–1.03) 0.288) |
|  | South | 1.14 (1.07–1.21) <0.001 | 1.01 (0.95–1.08) 0.749) |
|  | West | 1.00 (0.93–1.08) 0.958 | 0.92 (0.85–0.99) 0.033) |
| Hospital distance (miles) | <12.5 | — | — |
|  | 12.5–49.9 | 1.10 (1.05–1.16) <0.001 | 1.06 (1.00–1.12) 0.041 |
|  | ≥50 | 1.13 (1.07–1.20) <0.001 | 1.13 (1.05–1.21) 0.001 |
| Year of diagnosis | 2010–2011 | — | — |
|  | 2012–2013 | 0.95 (0.90–1.00) 0.065 | 0.95 (0.90–1.00) 0.071 |
|  | 2014–2015 | 0.92 (0.86–0.99) 0.019 | 0.93 (0.87–1.00) 0.057 |
|  | 2016–2017 | 0.87 (0.81–0.92) <0.001 | 0.88 (0.83–0.94) <0.001 |
| Age at diagnosis (years) | 18–35 | — | — |
|  | 36–50 | 1.06 (0.77–1.45) 0.725 | 1.02 (0.73–1.42) 0.916 |
|  | 51–65 | 1.21 (0.89–1.64) 0.231 | 1.16 (0.84–1.61) 0.373 |
|  | 66–80 | 1.45 (1.07–1.97) 0.018 | 1.34 (0.97–1.87) 0.080 |
|  | 80+ | 2.30 (1.65–3.20) <0.001 | 2.28 (1.61–3.25) <0.001 |
| Sex | Male | — | — |
|  | Female | 0.82 (0.77–0.87) <0.001 | 0.81 (0.76–0.86) <0.001 |
| Race | White | — | — |
|  | Other | 0.92 (0.85–1.00) 0.065 | 0.91 (0.83–1.00) 0.045 |
| CDCC score | 0 | — | — |
|  | 1 | 1.12 (1.06–1.18) <0.001 | 1.11 (1.06–1.17) <0.001 |
|  | 2 | 1.16 (1.05–1.28) 0.002 | 1.16 (1.06–1.28) 0.002 |
|  | 3+ | 1.33 (1.15–1.54) <0.001 | 1.32 (1.14–1.53) <0.001 |
| Insurance status | Medicare | — | — |
|  | Medicaid | 0.91 (0.83–1.00) 0.059 | 0.97 (0.87–1.08) 0.599 |
|  | Private | 0.77 (0.73–0.81) <0.001 | 0.86 (0.81–0.92) <0.001 |
|  | Not insured/other | 0.96 (0.86–1.08) 0.512 | 1.03 (0.92–1.16) 0.609 |
| Education level (%) | >21 | — | — |
|  | 13–20.9 | 1.53 (1.43–1.63) <0.001 | 1.32 (1.23–1.42) <0.001 |
|  | 7–12.9 | 1.36 (1.28–1.45) <0.001 | 1.34 (1.25–1.44) <0.001 |
|  | <7 | 1.26 (1.18–1.35) <0.001 | 1.43 (1.32–1.54) <0.001 |
| Medical income ($) | ≤47,999 | — | — |
|  | 48,000–62,999 | 0.92 (0.87–0.98) 0.005 | 0.88 (0.83–0.94) <0.001 |
|  | 63,000+ | 0.69 (0.66–0.73) <0.001 | 0.72 (0.67–0.77) <0.001 |
| Residence | Metro | — | — |
|  | Urban | 1.15 (1.09–1.22) <0.001 | 1.00 (0.94–1.07) 0.951 |
|  | Rural | 0.74 (0.67–0.81) <0.001 | 0.72 (0.66–0.80) <0.001 |
| AJCC clinical T stage | cT1 | — | — |
|  | cT2 | 1.68 (1.55–1.83) <0.001 | 1.55 (1.41–1.70) <0.001 |
|  | cT3 | 2.16 (2.01–2.32) <0.001 | 1.90 (1.74–2.08) <0.001 |
|  | cT4 | 2.70 (2.33–3.14) <0.001 | 2.32 (1.98–2.72) <0.001 |
|  | cTx | 2.05 (1.88–2.24) <0.001 | 1.77 (1.60–1.96) <0.001 |
| AJCC clinical N stage | cN0 | — | — |
|  | cN1 | 1.42 (1.35–1.49) <0.001 | 1.23 (1.17–1.30) <0.001 |
|  | cN2 | 1.51 (1.40–1.63) <0.001 | 1.37 (1.26–1.48) <0.001 |
|  | cN3 | 2.06 (1.78–2.40) <0.001 | 1.75 (1.50–2.04) <0.001 |
|  | cNx | 1.40 (1.27–1.53) <0.001 | 1.15 (1.03–1.28) 0.014 |
| Histology | Adenocarcinoma | — | — |
|  | SCC | 1.07 (1.01–1.13) 0.016 | 1.12 (1.05–1.19) 0.001 |
| Neoadjuvant therapy | None | — | — |
|  | NCRT | 1.44 (1.37–1.52) <0.001 | 1.04 (0.97–1.12) 0.232 |
|  | NAC | 1.47 (1.36–1.59) <0.001 | 1.08 (0.99–1.18) 0.083 |
| Textbook outcome | No | — | — |
|  | Yes | 0.61 (0.58–0.64) <0.001 | 0.64 (0.60–0.67) <0.001 |
| Surgical approach | Open | — | — |
|  | Laparoscopic | 0.86 (0.82–0.91) <0.001 | 0.96 (0.91–1.01) 0.108 |
|  | Robotic | 0.85 (0.78–0.93) <0.001 | 0.92 (0.84–1.00) 0.049 |

HR, hazard ratio; CDCC,; AJCC, American Joint Committee on Cancer; SCC, squamous cell carcinoma; NCRT, n**eoadjuvant chemoradiotherapy**; NAC, n**eoadjuvant chemotherapy**

**TABLE S4** Textbook outcomes and long-term survival for patients undergoing esophagectomy for esophageal cancer or gastrectomy for gastric cancer by surgical approach in a sensitivity analysis

|  |  | Textbook outcomes,  *n* (%) | Adjusted OR (95 % CI) | *p* Value | Adjusted HR (95 % CI) | *p* Value |
| --- | --- | --- | --- | --- | --- | --- |
| All **p**atients | Esophagectomy |  |  |  |  |  |
|  | Open | 2428 (21.2) | Reference |  | Reference |  |
|  | Laparoscopic | 1517 (31.4) | 1.45 (1.34–1.57) | <0.001 | 0.95 (0.90–1.01) | 0.1 |
|  | Robotic | 1517 (31.4) | 1.64 (1.46–1.85) | <0.001 | 0.91 (0.84–1.00) | 0.048 |
|  | Gastrectomy |  |  |  |  |  |
|  | Open | 6567 (29.6) | Reference |  | Reference |  |
|  | Laparoscopic | 2446 (38.5) | 1.26 (1.18–1.34) | <0.001 | 0.90 (0.86–0.94) | <0.001 |
|  | Robotic | 733 (42.7) | 1.44 (1.29–1.60) | <0.001 | 0.90 (0.82–0.98) | 0.019 |

**TABLE S5** Baseline clinicopathologic characteristics of patients undergoing esophagectomy for esophageal cancer by surgical approach in high-volume (Quintile 5) centers

|  |  | Open  *n* (%) | Laparoscopic  *n* (%) | Robotic  *n* (%) | *p* Value |
| --- | --- | --- | --- | --- | --- |
| Facility type | Integrated | 50 (1.9) | 18 (1.5) | 5 (1.1) | 0.382 |
|  | Academic | 2534 (98.1) | 1159 (98.5) | 445 (98.9) |  |
| Facility location | Northeast | 489 (18.9) | 508 (43.2) | 121 (26.9) | <0.001 |
|  | Midwest | 1267 (49.0) | 318 (27.0) | 88 (19.6) |  |
|  | South | 716 (27.7) | 212 (18.0) | 236 (52.4) |  |
|  | West | 112 (4.3) | 139 (11.8) | 5 (1.1) |  |
| Hospital distance (miles) | <12.5 | 635 (24.6) | 359 (30.5) | 130 (28.9) | <0.001 |
|  | 12.5–49.9 | 612 (23.7) | 328 (27.9) | 119 (26.4) |  |
|  | ≥50 | 1337 (51.7) | 490 (41.6) | 201 (44.7) |  |
| Year of diagnosis | 2010–2011 | 790 (30.6) | 274 (23.3) | 65 (14.4) | <0.001 |
|  | 2012–2013 | 700 (27.1) | 298 (25.3) | 131 (29.1) |  |
|  | 2014–2015 | 291 (11.3) | 149 (12.7) | 80 (17.8) |  |
|  | 2016–2017 | 803 (31.1) | 456 (38.7) | 174 (38.7) |  |
| Age at diagnosis (years) | 18–35 | 26 (1.0) | 7 (0.6) | 2 (0.4) | 0.283 |
|  | 36–50 | 237 (9.2) | 108 (9.2) | 34 (7.6) |  |
|  | 51–65 | 1252 (48.5) | 533 (45.4) | 214 (47.6) |  |
|  | 66–80 | 1011 (39.2) | 505 (43.0) | 188 (41.8) |  |
|  | 80+ | 54 (2.1) | 21 (1.8) | 12 (2.7) |  |
| Sex | Male | 2154 (83.4) | 967 (82.2) | 372 (82.7) | 0.653 |
|  | Female | 430 (16.6) | 210 (17.8) | 78 (17.3) |  |
| Race | White | 2435 (94.2) | 1106 (94.0) | 418 (92.9) | 0.538 |
|  | Other | 149 (5.8) | 71 (6.0) | 32 (7.1) |  |
| CDCC score | 0 | 1845 (71.4) | 810 (68.8) | 337 (74.9) | 0.035 |
|  | 1–2 | 692 (26.8) | 352 (29.9) | 103 (22.9) |  |
|  | 2+ | 47 (1.8) | 15 (1.3) | 10 (2.2) |  |
| Insurance status | Medicare | 1056 (41.7) | 514 (44.9) | 207 (47.2) | <0.001 |
|  | Medicaid | 109 (4.3) | 88 (7.7) | 16 (3.6) |  |
|  | Private | 1171 (46.3) | 505 (44.1) | 200 (45.6) |  |
|  | Not Insured / Other | 194 (7.7) | 39 (3.4) | 16 (3.6) |  |
| Education level (%) | >21 | 590 (22.8) | 267 (22.7) | 122 (27.1) | 0.197 |
|  | 13–20.9 | 575 (22.3) | 244 (20.7) | 103 (22.9) |  |
|  | 7–12.9 | 834 (32.3) | 397 (33.7) | 143 (31.8) |  |
|  | <7 | 585 (22.6) | 269 (22.9) | 82 (18.2) |  |
| Medical income ($) | ≤47,999 | 936 (36.2) | 430 (36.5) | 160 (35.6) | 0.403 |
|  | 48,000–62,999 | 640 (24.8) | 270 (22.9) | 97 (21.6) |  |
|  | 63,000+ | 1008 (39.0) | 477 (40.5) | 193 (42.9) |  |
| Residence | Metro | 1667 (64.5) | 822 (69.8) | 342 (76.0) | <0.001 |
|  | Urban | 578 (22.4) | 248 (21.1) | 56 (12.4) |  |
|  | Rural | 339 (13.1) | 107 (9.1) | 52 (11.6) |  |
| AJCC clinical T stage | cT1 | 483 (18.7) | 235 (20.0) | 74 (16.4) | 0.115 |
|  | cT2 | 408 (15.8) | 218 (18.5) | 73 (16.2) |  |
|  | cT3 | 1414 (54.7) | 604 (51.3) | 262 (58.2) |  |
|  | cT4 | 50 (1.9) | 28 (2.4) | 11 (2.4) |  |
|  | cTx | 229 (8.9) | 92 (7.8) | 30 (6.7) |  |
| AJCC clinical N stage | cN0 | 1137 (44.0) | 520 (44.2) | 201 (44.7) | 0.008 |
|  | cN1 | 968 (37.5) | 443 (37.6) | 185 (41.1) |  |
|  | cN2 | 305 (11.8) | 155 (13.2) | 56 (12.4) |  |
|  | cN3 | 76 (2.9) | 25 (2.1) | 3 (0.7) |  |
|  | cNx | 98 (3.8) | 34 (2.9) | 5 (1.1) |  |
| Histology | Adenocarcinoma | 2169 (83.9) | 964 (81.9) | 364 (80.9) | 0.132 |
|  | SCC | 415 (16.1) | 213 (18.1) | 86 (19.1) |  |
| Neoadjuvant therapy | None | 731 (28.3) | 324 (27.5) | 102 (22.7) | <0.001 |
|  | NCRT | 1612 (62.4) | 735 (62.4) | 328 (72.9) |  |
|  | NAC | 241 (9.3) | 118 (10.0) | 20 (4.4) |  |
| Tumor grade | Well | 111 (4.3) | 62 (5.3) | 34 (7.6) | <0.001 |
|  | Moderate | 1037 (40.1) | 525 (44.6) | 197 (43.8) |  |
|  | Poor | 1029 (39.8) | 454 (38.6) | 167 (37.1) |  |
|  | Anaplastic | 407 (15.8) | 136 (11.6) | 52 (11.6) |  |
| AJCC pathologic T stage | pT0 | 496 (19.2) | 236 (20.1) | 121 (26.9) | 0.001 |
|  | pT1 | 743 (28.8) | 351 (29.8) | 133 (29.6) |  |
|  | pT2 | 391 (15.1) | 170 (14.4) | 58 (12.9) |  |
|  | pT3 | 760 (29.4) | 351 (29.8) | 126 (28.0) |  |
|  | pT4 | 35 (1.4) | 14 (1.2) | 1 (0.2) |  |
|  | pTx | 159 (6.2) | 55 (4.7) | 11 (2.4) |  |
| AJCC pathologic N stage | pN0 | 1648 (63.8) | 731 (62.1) | 303 (67.3) | 0.005 |
|  | pN1 | 490 (19.0) | 232 (19.7) | 91 (20.2) |  |
|  | pN2 | 217 (8.4) | 124 (10.5) | 39 (8.7) |  |
|  | pN3 | 97 (3.8) | 48 (4.1) | 7 (1.6) |  |
|  | pNx | 132 (5.1) | 42 (3.6) | 10 (2.2) |  |
| Lymphovascular invasion | Absent | 1315 (50.9) | 681 (57.9) | 235 (52.2) | <0.001 |
|  | Present | 419 (16.2) | 233 (19.8) | 77 (17.1) |  |
|  | Unknown | 850 (32.9) | 263 (22.3) | 138 (30.7) |  |
| 30-Day mortality | No | 2534 (98.1) | 1151 (97.8) | 439 (97.6) | 0.720 |
|  | Yes | 50 (1.9) | 26 (2.2) | 11 (2.4) |  |

CDCC,; AJCC, American Joint Committee on Cancer; SCC, squamous cell carcinoma; NCRT, n**eoadjuvant chemoradiotherapy**; NAC, n**eoadjuvant chemotherapy**

**TABLE S6** Individual textbook parameters of patients undergoing esophagectomy for esophageal cancer or gastrectomy for gastric cancer by surgical approach in high-volume (Quintile 5) centers

|  |  | Open  *n* (%) | Laparoscopic  *n* (%) | Robotic  *n* (%) | *p* Value |
| --- | --- | --- | --- | --- | --- |
| Esophagectomy |  |  |  |  |  |
| Regional nodes examined | <15 | 1234 (47.8) | 387 (32.9) | 135 (30.0) | <0.001 |
|  | >15 | 1350 (52.2) | 790 (67.1) | 315 (70.0) |  |
| Margin status | Negative | 2466 (95.4) | 1106 (94.0) | 435 (96.7) | 0.044 |
|  | Positive | 118 (4.6) | 71 (6.0) | 15 (3.3) |  |
| Length of stay (days) | **≤**21 | 309 (12.0) | 132 (11.2) | 49 (10.9) | 0.702 |
|  | >21 | 2275 (88.0) | 1045 (88.8) | 401 (89.1) |  |
| 90-Day mortality | No | 2454 (95.0) | 1114 (94.6) | 426 (94.7) | 0.903 |
|  | Yes | 130 (5.0) | 63 (5.4) | 24 (5.3) |  |
| 30-Day readmission | No | 2387 (92.4) | 1095 (93.1) | 417 (92.9) | 0.756 |
|  | Yes–unplanned | 11 (0.4) | 7 (0.6) | 3 (0.7) |  |
|  | Yes–planned | 186 (7.2) | 74 (6.3) | 29 (6.5) |  |
|  |  |  |  |  |  |
| Gastrectomy |  |  |  |  |  |
| Regional nodes examined | <15 | 1557 (34.9) | 522 (28.1) | 119 (21.5) | <0.001 |
|  | >15 | 2902 (65.1) | 1333 (71.9) | 435 (78.5) |  |
| Margin status | Negative | 4105 (92.1) | 1752 (94.4) | 521 (94.0) | 0.002 |
|  | Positive | 354 (7.9) | 103 (5.6) | 33 (6.0) |  |
| Length of stay | <21 | 406 (9.1) | 152 (8.2) | 47 (8.5) | 0.488 |
|  | >21 | 4053 (90.9) | 1703 (91.8) | 507 (91.5) |  |
| 90-Day mortality | No | 4256 (95.4) | 1782 (96.1) | 533 (96.2) | 0.445 |
|  | Yes | 203 (4.6) | 73 (3.9) | 21 (3.8) |  |
| 30-Day readmission | No | 4068 (91.4) | 1741 (94.0) | 521 (94.0) | <0.001 |
|  | Yes–unplanned | 75 (1.7) | 12 (0.6) | 2 (0.4) |  |
|  | Yes–planned | 310 (7.0) | 99 (5.3) | 31 (5.6) |  |

**TABLE S7** Logistic regression of factors associated with textbook outcome for patients undergoing esophagectomy for esophageal cancer in high-volume centers

|  |  | OR (univariable) *p* Value | OR (multivariable) *p* Value |
| --- | --- | --- | --- |
| Facility type | Integrated | — | — |
|  | Academic | 1.20 (0.75–1.93) 0.444 | 4.29 (1.87–10.08) 0.001 |
| Facility location | Northeast | — | — |
|  | Midwest | 0.31 (0.27–0.36) <0.001 | 0.32 (0.27–0.38) <0.001 |
|  | South | 0.41 (0.35–0.48) <0.001 | 0.41 (0.33–0.50) <0.001 |
|  | West | 0.94 (0.71–1.25) 0.669 | 1.39 (0.97–2.03) 0.079 |
| Hospital distance (miles) | <12.5 | — | — |
|  | 12.5–49.9 | 1.17 (0.98–1.38) 0.075 | 1.05 (0.85–1.30) 0.659 |
|  | ≥50 | 0.93 (0.80–1.08) 0.336 | 1.16 (0.93–1.44) 0.189 |
| Year of diagnosis | 2010–2011 | — | — |
|  | 2012–2013 | 1.35 (1.14–1.59) <0.001 | 1.32 (1.10–1.58) 0.003 |
|  | 2014–2015 | 1.62 (1.31–2.00) <0.001 | 1.63 (1.30–2.04) <0.001 |
|  | 2016–2017 | 1.75 (1.49–2.05) <0.001 | 1.80 (1.52–2.15) <0.001 |
| Age at diagnosis (years) | 18–35 | — | — |
|  | 36–50 | 1.60 (0.79–3.35) 0.199 | 1.10 (0.40–3.06) 0.848 |
|  | 51–65 | 1.52 (0.77–3.12) 0.234 | 1.02 (0.36–2.92) 0.966 |
|  | 66–80 | 1.47 (0.75–3.03) 0.271 | 1.00 (0.35–2.88) 0.996 |
|  | 80+ | 1.03 (0.46–2.37) 0.935 | 0.67 (0.21–2.11) 0.497 |
| Sex | Male | — | — |
|  | Female | 0.98 (0.83–1.15) 0.788 | 1.09 (0.91–1.31) 0.343 |
| Race | White | — | — |
|  | Other | 0.96 (0.75–1.24) 0.776 | 1.12 (0.83–1.50) 0.449 |
| CDCC score | 0 | — | — |
|  | 1–2 | 1.05 (0.92–1.20) 0.478 | 0.95 (0.82–1.11) 0.543 |
|  | 2+ | 0.68 (0.42–1.10) 0.123 | 0.70 (0.41–1.16) 0.172 |
| Insurance status | Medicare | — | — |
|  | Medicaid | 0.85 (0.64–1.14) 0.284 | 0.92 (0.66–1.28) 0.604 |
|  | Private | 1.19 (1.05–1.36) 0.008 | 1.17 (0.98–1.41) 0.082 |
|  | Not Insured / Other | 0.56 (0.42–0.74) <0.001 | 0.63 (0.46–0.87) 0.005 |
| Education level (%) | >21 | — | — |
|  | 13–20.9 | 1.16 (0.96–1.39) 0.119 | 1.22 (0.97–1.53) 0.096 |
|  | 7–12.9 | 1.32 (1.12–1.56) 0.001 | 1.26 (1.01–1.57) 0.037 |
|  | <7 | 1.51 (1.26–1.81) <0.001 | 1.35 (1.07–1.71) 0.011 |
| Medical income ($) | ≤47,999 | — | — |
|  | 48,000–62,999 | 0.98 (0.83–1.14) 0.757 | 0.87 (0.72–1.05) 0.148 |
|  | 63,000+ | 1.23 (1.07–1.41) 0.004 | 1.01 (0.82–1.24) 0.925 |
| Residence | Metro | — | — |
|  | Urban | 0.70 (0.60–0.81) <0.001 | 0.83 (0.70–1.00) 0.051 |
|  | Rural | 0.99 (0.82–1.20) 0.945 | 1.06 (0.86–1.31) 0.593 |
| AJCC clinical T stage | cT1 | — | — |
|  | cT2 | 1.22 (1.00–1.50) 0.052 | 1.34 (1.05–1.72) 0.021 |
|  | cT3 | 1.15 (0.98–1.36) 0.084 | 1.20 (0.94–1.52) 0.140 |
|  | cT4 | 0.96 (0.62–1.49) 0.867 | 0.91 (0.55–1.50) 0.723 |
|  | cTx | 0.74 (0.57–0.96) 0.024 | 0.73 (0.53–0.99) 0.044 |
| AJCC clinical N stage | cN0 | — | — |
|  | cN1 | 1.04 (0.91–1.18) 0.606 | 0.96 (0.81–1.13) 0.601 |
|  | cN2 | 1.09 (0.89–1.32) 0.398 | 0.96 (0.76–1.21) 0.726 |
|  | cN3 | 0.83 (0.55–1.23) 0.359 | 0.78 (0.50–1.20) 0.257 |
|  | cNx | 0.52 (0.35–0.75) 0.001 | 0.91 (0.58–1.42) 0.684 |
| Histology | Adenocarcinoma | — | — |
|  | SCC | 0.70 (0.60–0.83) <0.001 | 0.63 (0.52–0.77) <0.001 |
| Neoadjuvant therapy | None | — | — |
|  | NCRT | 1.13 (0.98–1.30) 0.083 | 0.86 (0.70–1.06) 0.163 |
|  | NAC | 1.16 (0.92–1.46) 0.216 | 0.87 (0.65–1.16) 0.349 |
| Surgical approach | Open | — | — |
|  | Laparoscopic | 1.67 (1.46–1.92) <0.001 | 1.15 (0.98–1.35) 0.080 |
|  | Robotic | 2.01 (1.64–2.47) <0.001 | 1.72 (1.37–2.15) <0.001 |

OR, odds ratio; CDCC,; AJCC, American Joint Committee on Cancer; SCC, squamous cell carcinoma; NCRT, n**eoadjuvant chemoradiotherapy**; NAC, n**eoadjuvant chemotherapy**

**TABLE S8** Cox regression of factors associated with long-term survival of patients undergoing esophagectomy for esophageal cancer in high-volume (Quintile 5) centers

|  |  | HR (univariable) *p* Value | HR (multivariable) *p* Value |
| --- | --- | --- | --- |
| Facility type | Integrated | — | — |
|  | Academic | 1.36 (0.90–2.06) 0.139 | 0.89 (0.45–1.73) 0.724 |
| Facility location | Northeast | — | — |
|  | Midwest | 1.09 (0.97–1.23) 0.135 | 0.88 (0.77–1.01) 0.061 |
|  | South | 1.13 (0.99–1.28) 0.062 | 0.85 (0.73–0.98) 0.030 |
|  | West | 0.88 (0.70–1.10) 0.246 | 0.63 (0.48–0.82) 0.001 |
| Hospital distance (miles) | <12.5 | — | — |
|  | 12.5–49.9 | 1.75 (1.53–2.01) <0.001 | 1.36 (1.16–1.60) <0.001 |
|  | ≥50 | 1.90 (1.67–2.15) <0.001 | 1.52 (1.29–1.80) <0.001 |
| Year of diagnosis | 2010–2011 | — | — |
|  | 2012–2013 | 0.96 (0.85–1.07) 0.429 | 0.99 (0.88–1.11) 0.882 |
|  | 2014–2015 | 0.95 (0.82–1.11) 0.514 | 1.06 (0.91–1.24) 0.447 |
|  | 2016–2017 | 0.92 (0.80–1.06) 0.239 | 0.99 (0.86–1.15) 0.931 |
| Age at diagnosis (years) | 18–35 | — | — |
|  | 36–50 | 1.18 (0.64–2.18) 0.593 | 0.89 (0.38–2.06) 0.783 |
|  | 51–65 | 1.24 (0.68–2.24) 0.482 | 0.92 (0.39–2.18) 0.857 |
|  | 66–80 | 1.45 (0.80–2.63) 0.219 | 1.07 (0.45–2.53) 0.885 |
|  | 80+ | 2.27 (1.19–4.35) 0.013 | 1.88 (0.76–4.64) 0.172 |
| Sex | Male | — | — |
|  | Female | 0.77 (0.68–0.88) <0.001 | 0.79 (0.69–0.90) 0.001 |
| Race | White | — | — |
|  | Other | 0.79 (0.64–0.98) 0.030 | 0.85 (0.68–1.07) 0.174 |
| CDCC score | 0 | — | — |
|  | 1–2 | 1.19 (1.08–1.32) 0.001 | 1.19 (1.07–1.33) 0.001 |
|  | 2+ | 1.60 (1.14–2.24) 0.006 | 1.66 (1.18–2.34) 0.003 |
| Insurance status | Medicare | — | — |
|  | Medicaid | 0.93 (0.75–1.15) 0.509 | 1.00 (0.79–1.27) 0.995 |
|  | Private | 0.82 (0.75–0.91) <0.001 | 0.87 (0.76–1.00) 0.042 |
|  | Not Insured / Other | 0.79 (0.64–0.97) 0.027 | 0.90 (0.71–1.14) 0.388 |
| Education level (%) | >21 | — | — |
|  | 13–20.9 | 2.07 (1.78–2.40) <0.001 | 1.54 (1.29–1.84) <0.001 |
|  | 7–12.9 | 1.83 (1.59–2.10) <0.001 | 1.54 (1.30–1.83) <0.001 |
|  | <7 | 1.75 (1.50–2.03) <0.001 | 1.70 (1.41–2.04) <0.001 |
| Medical income ($) | ≤47,999 | — | — |
|  | 48,000–62,999 | 0.88 (0.78–0.98) 0.023 | 0.82 (0.72–0.93) 0.002) |
|  | 63,000+ | 0.60 (0.54–0.67) <0.001 | 0.69 (0.59–0.80) <0.001 |
| Residence | Metro | — | — |
|  | Urban | 1.25 (1.12–1.40) <0.001 | 1.03 (0.90–1.16) 0.698 |
|  | Rural | 0.57 (0.47–0.67) <0.001 | 0.49 (0.41–0.59) <0.001 |
| AJCC clinical T stage | cT1 | — | — |
|  | cT2 | 1.65 (1.38–1.96) <0.001 | 1.51 (1.23–1.84) <0.001 |
|  | cT3 | 2.30 (1.99–2.65) <0.001 | 2.04 (1.68–2.48) <0.001 |
|  | cT4 | 3.37 (2.50–4.53) <0.001 | 3.04 (2.20–4.20) <0.001 |
|  | cTx | 2.01 (1.64–2.47) <0.001 | 1.84 (1.45–2.33) <0.001 |
| AJCC clinical N stage | cN0 | — | — |
|  | cN1 | 1.51 (1.36–1.68) <0.001 | 1.21 (1.07–1.36) 0.002 |
|  | cN2 | 1.53 (1.32–1.77) <0.001 | 1.32 (1.12–1.56) 0.001 |
|  | cN3 | 2.59 (1.99–3.38) <0.001 | 2.02 (1.53–2.67) <0.001 |
|  | cNx | 1.34 (1.03–1.75) 0.028 | 1.20 (0.88–1.63) 0.257 |
| Histology | Adenocarcinoma | — | — |
|  | SCC | 0.94 (0.83–1.07) 0.342 | 0.99 (0.86–1.14) 0.876 |
| Neoadjuvant therapy | None | — | — |
|  | NCRT | 1.57 (1.40–1.76) <0.001 | 1.00 (0.86–1.17) 0.978 |
|  | NAC | 1.60 (1.34–1.91) <0.001 | 1.07 (0.86–1.32) 0.550 |
| Textbook outcome | No | — | — |
|  | Yes | 0.61 (0.56–0.67) <0.001 | 0.58 (0.52–0.64) <0.001 |
| Surgical approach | Open | — | — |
|  | Laparoscopic | 0.94 (0.84–1.04) 0.240 | 0.98 (0.87–1.10) 0.693 |
|  | Robotic | 0.78 (0.66–0.92) 0.003 | 0.81 (0.68–0.96) 0.017 |

HR, hazard ratio; CDCC,; AJCC, American Joint Committee on Cancer; SCC, squamous cell carcinoma; NCRT, n**eoadjuvant chemoradiotherapy**; NAC, n**eoadjuvant chemotherapy**

**TABLE S9** Logistic regression of factors associated with textbook outcome for patients undergoing gastrectomy for gastric cancer

|  |  | OR (univariable) *p* Value | OR (multivariable) *p* Value |
| --- | --- | --- | --- |
| Center volume | 1 (lowest) | — | — |
|  | 2 | 1.25 (1.15–1.37) <0.001 | 1.15 (1.05–1.26) 0.003 |
|  | 3 | 1.67 (1.54–1.82) <0.001 | 1.54 (1.41–1.69) <0.001 |
|  | 4 | 2.62 (2.41–2.85) <0.001 | 2.13 (1.94–2.34) <0.001 |
|  | 5 (highest) | 3.45 (3.17–3.75) <0.001 | 2.58 (2.32–2.87) <0.001 |
| Facility type | Community | — | — |
|  | Integrated | 1.33 (1.24–1.43) <0.001 | 1.03 (0.95–1.11) 0.491 |
|  | Academic | 2.07 (1.97–2.18) <0.001 | 1.15 (1.07–1.23) <0.001 |
| Facility location | Northeast | — | — |
|  | Midwest | 0.63 (0.59–0.68) <0.001 | 0.81 (0.75–0.87) <0.001 |
|  | South | 0.54 (0.51–0.58) <0.001 | 0.68 (0.63–0.72) <0.001 |
|  | West | 0.80 (0.74–0.85) <0.001 | 0.98 (0.90–1.06) 0.553 |
| Hospital distance (miles) | <12.5 | — | — |
|  | 12.5–49.9 | 1.02 (0.97–1.07) 0.477) | 0.99 (0.93–1.05) 0.683 |
|  | ≥50 | 1.17 (1.10–1.25) <0.001 | 1.02 (0.94–1.11) 0.640 |
| Year of diagnosis | 2010–2011 | — | — |
|  | 2012–2013 | 1.15 (1.08–1.23) <0.001 | 1.14 (1.06–1.22) <0.001 |
|  | 2014–2015 | 1.38 (1.28–1.50) <0.001 | 1.33 (1.22–1.44) <0.001 |
|  | 2016–2017 | 1.67 (1.57–1.78) <0.001 | 1.56 (1.46–1.66) <0.001 |
| Age at diagnosis, (years) | 18–35 | — | — |
|  | 36–50 | 0.89 (0.71–1.12) 0.314 | 1.00 (0.77–1.28) 0.974 |
|  | 51–65 | 0.83 (0.67–1.03) 0.096 | 0.99 (0.77–1.26) 0.913 |
|  | 66–80 | 0.71 (0.57–0.88) 0.002 | 0.92 (0.72–1.17) 0.482 |
|  | 80+ | 0.47 (0.38–0.59) <0.001 | 0.68 (0.53–0.88) 0.003 |
| Sex | Male | — | — |
|  | Female | 1.03 (0.98–1.09) 0.195 | 1.09 (1.03–1.15) 0.002 |
| Race | White | — | — |
|  | Other | 1.35 (1.28–1.42) <0.001 | 1.33 (1.26–1.41) <0.001 |
| CDCC score | 0 | — | — |
|  | 1–2 | 0.82 (0.78–0.86) <0.001 | 0.91 (0.86–0.96) <0.001 |
|  | 2+ | 0.75 (0.66–0.85) <0.001 | 0.83 (0.73–0.95) 0.007 |
| Insurance status | Medicare | — | — |
|  | Medicaid | 1.31 (1.20–1.43) <0.001 | 1.00 (0.90–1.11) 0.965 |
|  | Private | 1.36 (1.29–1.43) <0.001 | 1.13 (1.05–1.21) 0.001 |
|  | Not Insured / Other | 0.93 (0.82–1.04) 0.206 | 0.87 (0.77–0.99) 0.038 |
| Education level (%) | >21 | — | — |
|  | 13–20.9 | 0.84 (0.79–0.90) <0.001 | 0.93 (0.86–0.99) 0.028 |
|  | 7–12.9 | 0.88 (0.83–0.93) <0.001 | 0.89 (0.83–0.95) 0.001 |
|  | <7 | 0.98 (0.92–1.05) 0.629 | 0.91 (0.84–0.99) 0.028 |
| Medical income ($) | ≤47,999 | — | — |
|  | 48,000–62,999 | 1.11 (1.04–1.18) 0.001 | 1.09 (1.02–1.17) 0.010 |
|  | 63,000+ | 1.32 (1.25–1.39) <0.001 | 1.14 (1.07–1.22) <0.001 |
| Residence | Metro | — | — |
|  | Urban | 0.80 (0.75–0.86) <0.001 | 0.94 (0.86–1.02) 0.134 |
|  | Rural | 1.20 (1.08–1.33) 0.001 | 1.18 (1.05–1.33) 0.004 |
| AJCC clinical T stage | cT1 | — | — |
|  | cT2 | 1.24 (1.14–1.35) <0.001 | 1.20 (1.09–1.31) <0.001 |
|  | cT3 | 1.27 (1.19–1.36) <0.001 | 1.18 (1.09–1.28) <0.001 |
|  | cT4 | 0.86 (0.77–0.96) 0.009 | 0.82 (0.73–0.93) 0.002 |
|  | cTx | 0.90 (0.84–0.96) 0.002 | 1.06 (0.98–1.15) 0.160 |
| AJCC clinical N stage | cN0 | — | — |
|  | cN1 | 1.26 (1.18–1.33) <0.001 | 1.12 (1.04–1.20) 0.001 |
|  | cN2 | 1.22 (1.12–1.33) <0.001 | 1.10 (1.00–1.22) 0.058 |
|  | cN3 | 1.17 (1.02–1.35) 0.029 | 1.34 (1.14–1.57) <0.001 |
|  | cNx | 0.82 (0.77–0.88) <0.001 | 0.93 (0.85–1.00) 0.065 |
| Neoadjuvant therapy | None | — | — |
|  | NCRT | 1.08 (1.01–1.14) 0.016 | 0.75 (0.70–0.82) <0.001 |
|  | NAC | 1.84 (1.73–1.95) <0.001 | 1.31 (1.22–1.41) <0.001 |
| Surgical approach | Open | — | — |
|  | Laparoscopic | 1.43 (1.36–1.52) <0.001 | 1.19 (1.12–1.26) <0.001 |
|  | Robotic | 1.67 (1.51–1.84) <0.001 | 1.30 (1.17–1.45) <0.001 |

OR, odds ratio; CDCC,; AJCC, American Joint Committee on Cancer; NCRT, n**eoadjuvant chemoradiotherapy**; NAC, n**eoadjuvant chemotherapy**

**TABLE S10** Cox regression of factors associated with long-term survival of patients undergoing gastrectomy for gastric cancer

|  |  | HR (univariable) *p* Value | HR (multivariable) *p* Value |
| --- | --- | --- | --- |
| Center volume | 1 (lowest) | — | — |
|  | 2 | 0.90 (0.85–0.95) <0.001 | 1.00 (0.95–1.06) 0.997 |
|  | 3 | 0.81 (0.77–0.86) <0.001 | 0.94 (0.89–1.00) 0.043 |
|  | 4 | 0.69 (0.65–0.72) <0.001 | 0.89 (0.83–0.94) <0.001 |
|  | 5 (highest) | 0.57 (0.54–0.61) <0.001 | 0.82 (0.76–0.88) <0.001 |
| Facility type | Community | — | — |
|  | Integrated | 0.91 (0.86–0.95) <0.001 | 1.10 (1.04–1.15) 0.001 |
|  | Academic | 0.70 (0.68–0.73) <0.001 | 0.97 (0.92–1.02) 0.228 |
| Facility location | Northeast | — | — |
|  | Midwest | 1.26 (1.20–1.33) <0.001 | 1.07 (1.01–1.13) 0.013 |
|  | South | 1.33 (1.27–1.39) <0.001 | 1.14 (1.09–1.20) <0.001 |
|  | West | 1.17 (1.11–1.23) <0.001 | 1.09 (1.03–1.15) 0.003 |
| Hospital distance (miles) | <12.5 | — | — |
|  | 12.5–49.9 | 1.06 (1.02–1.10) 0.004 | 1.06 (1.02–1.10) 0.007 |
|  | ≥50 | 1.08 (1.03–1.14) 0.001 | 1.07 (1.01–1.14) 0.023 |
| Year of diagnosis | 2010–2011 | — | — |
|  | 2012–2013 | 0.95 (0.91–0.99) 0.013 | 1.01 (0.96–1.05) 0.781 |
|  | 2014–2015 | 0.88 (0.84–0.93) <0.001 | 1.00 (0.94–1.05) 0.917 |
|  | 2016–2017 | 0.81 (0.77–0.85) <0.001 | 0.94 (0.89–0.99) 0.019 |
| Age at diagnosis (years) | 18–35 | — | — |
|  | 36–50 | 0.85 (0.72–1.02) 0.073 | 0.96 (0.80–1.16) 0.700 |
|  | 51–65 | 0.86 (0.73–1.01) 0.071 | 0.97 (0.81–1.16) 0.730 |
|  | 66–80 | 1.08 (0.91–1.27) 0.384 | 1.12 (0.93–1.34) 0.224 |
|  | 80+ | 1.72 (1.45–2.03) <0.001 | 1.70 (1.41–2.04) <0.001 |
| Sex | Male | — | — |
|  | Female | 0.95 (0.92–0.99) 0.013 | 0.92 (0.89–0.96) <0.001 |
| Race | White | — | — |
|  | Other | 0.79 (0.76–0.83) <0.001 | 0.84 (0.81–0.88) <0.001 |
| CDCC score | 0 | — | — |
|  | 1–2 | 1.14 (1.10–1.18) <0.001 | 1.08 (1.04–1.12) <0.001 |
|  | 2+ | 1.41 (1.29–1.53) <0.001 | 1.30 (1.19–1.42) <0.001 |
| Insurance status | Medicare | — | — |
|  | Medicaid | 0.75 (0.70–0.80) <0.001 | 0.89 (0.82–0.96) 0.003 |
|  | Private | 0.72 (0.69–0.75) <0.001 | 0.86 (0.82–0.90) <0.001 |
|  | Not Insured / Other | 0.80 (0.73–0.87) <0.001 | 0.84 (0.77–0.92) <0.001 |
| Education level (%) | >21 | — | — |
|  | 13–20.9 | 1.35 (1.29–1.41) <0.001 | 1.24 (1.18–1.30) <0.001 |
|  | 7–12.9 | 1.29 (1.23–1.35) <0.001 | 1.31 (1.25–1.38) <0.001 |
|  | <7 | 1.19 (1.13–1.25) <0.001 | 1.40 (1.32–1.48) <0.001 |
| Medical income ($) | ≤47,999 | — | — |
|  | 48,000–62,999 | 0.91 (0.87–0.95) <0.001 | 0.87 (0.83–0.91) <0.001 |
|  | 63,000 + | 0.72 (0.69–0.75) <0.001 | 0.74 (0.70–0.77) <0.001 |
| Residence | Metro | — | — |
|  | Urban | 1.23 (1.17–1.29) <0.001 | 1.05 (0.99–1.11) 0.097 |
|  | Rural | 0.85 (0.79–0.93) <0.001 | 0.87 (0.80–0.95) 0.002) |
| AJCC clinical T stage | cT1 | — | — |
|  | cT2 | 1.43 (1.33–1.53) <0.001 | 1.42 (1.32–1.52) <0.001 |
|  | cT3 | 1.86 (1.75–1.96) <0.001 | 1.78 (1.67–1.90) <0.001 |
|  | cT4 | 3.18 (2.95–3.43) <0.001 | 2.70 (2.49–2.93) <0.001 |
|  | cTx | 2.10 (1.99–2.22) <0.001 | 1.84 (1.73–1.95) <0.001 |
| AJCC clinical N stage | cN0 | — | — |
|  | cN1 | 1.30 (1.24–1.36) <0.001 | 1.32 (1.26–1.39) <0.001 |
|  | cN2 | 1.52 (1.43–1.62) <0.001 | 1.51 (1.41–1.61) <0.001 |
|  | cN3 | 2.43 (2.23–2.65) <0.001 | 2.07 (1.88–2.27) <0.001 |
|  | cNx | 1.61 (1.54–1.69) <0.001 | 1.35 (1.28–1.42) <0.001 |
| Neoadjuvant therapy | None | — | — |
|  | NCRT | 1.03 (0.98–1.07) 0.210 | 0.95 (0.90–1.01) 0.100 |
|  | NAC | 0.86 (0.82–0.90) <0.001 | 0.90 (0.85–0.95) <0.001 |
| Textbook outcome | No | — | — |
|  | Yes | 0.53 (0.51–0.55) <0.001 | 0.58 (0.56–0.60) <0.001 |
| Surgical approach | Open | — | — |
|  | Laparoscopic | 0.76 (0.73–0.80) <0.001 | 0.89 (0.85–0.94) <0.001 |
|  | Robotic | 0.72 (0.66–0.79) <0.001 | 0.88 (0.81–0.96) 0.006 |

HR, hazard ratio; CDCC,; AJCC, American Joint Committee on Cancer; NCRT, n**eoadjuvant chemoradiotherapy**; NAC, n**eoadjuvant chemotherapy**

**TABLE S11** Baseline clinicopathologic characteristics of patients undergoing gastrectomy for gastric cancer by surgical approach in high-volume (Quintile 5) centers

|  |  | Open  *n* (%) | Laparoscopic *n* (%) | Robotic  *n* (%) | *p* Value |
| --- | --- | --- | --- | --- | --- |
| Facility type | Integrated | 261 (5.9) | 105 (5.7) | 33 (6.0) | 0.945 |
|  | Academic | 4198 (94.1) | 1750 (94.3) | 521 (94.0) |  |
| Facility location | Northeast | 1675 (37.6) | 988 (53.3) | 253 (45.7) | <0.001 |
|  | Midwest | 830 (18.6) | 315 (17.0) | 39 (7.0) |  |
|  | South | 1437 (32.2) | 350 (18.9) | 175 (31.6) |  |
|  | West | 517 (11.6) | 202 (10.9) | 87 (15.7) |  |
| Hospital distance (miles) | <12.5 | 1933 (43.4) | 863 (46.5) | 235 (42.4) | 0.106 |
|  | 12.5–49.9 | 1312 (29.4) | 536 (28.9) | 173 (31.2) |  |
|  | ≥50 | 1214 (27.2) | 456 (24.6) | 146 (26.4) |  |
| Year of diagnosis | 2010–2011 | 1346 (30.2) | 428 (23.1) | 60 (10.8) | <0.001 |
|  | 2012–2013 | 1156 (25.9) | 461 (24.9) | 116 (20.9) |  |
|  | 2014–2015 | 535 (12.0) | 247 (13.3) | 86 (15.5) |  |
|  | 2016–2017 | 1422 (31.9) | 719 (38.8) | 292 (52.7) |  |
| Age at diagnosis (years) | 18–35 | 47 (1.1) | 27 (1.5) | 6 (1.1) | 0.282 |
|  | 36–50 | 400 (9.0) | 163 (8.8) | 55 (9.9) |  |
|  | 51–65 | 1595 (35.9) | 655 (35.4) | 205 (37.1) |  |
|  | 66–80 | 1968 (44.2) | 842 (45.5) | 251 (45.4) |  |
|  | 80+ | 439 (9.9) | 165 (8.9) | 36 (6.5) |  |
| Sex | Male | 3131 (70.2) | 1348 (72.7) | 385 (69.5) | 0.115 |
|  | Female | 1328 (29.8) | 507 (27.3) | 169 (30.5) |  |
| Race | White | 3135 (70.3) | 1367 (73.7) | 396 (71.5) | 0.025 |
|  | Other | 1324 (29.7) | 488 (26.3) | 158 (28.5) |  |
| CDCC score | 0 | 3064 (68.7) | 1235 (66.6) | 365 (65.9) | 0.416 |
|  | 1–2 | 1266 (28.4) | 561 (30.2) | 171 (30.9) |  |
|  | 2+ | 129 (2.9) | 59 (3.2) | 18 (3.2) |  |
| Insurance status | Medicare | 2216 (50.3) | 925 (50.3) | 289 (52.6) | <0.001 |
|  | Medicaid | 319 (7.2) | 161 (8.8) | 33 (6.0) |  |
|  | Private | 1669 (37.9) | 706 (38.4) | 218 (39.7) |  |
|  | Not Insured / Other | 203 (4.6) | 47 (2.6) | 9 (1.6) |  |
| Education level (%) | >21 | 1192 (26.7) | 545 (29.4) | 174 (31.4) | 0.130 |
|  | 13–20.9 | 1016 (22.8) | 395 (21.3) | 127 (22.9) |  |
|  | 7–12.9 | 1271 (28.5) | 518 (27.9) | 144 (26.0) |  |
|  | <7 | 980 (22.0) | 397 (21.4) | 109 (19.7) |  |
| Medical income ($) | ≤47,999 | 1428 (32.0) | 613 (33.0) | 186 (33.6) | 0.437 |
|  | 48,000–62,999 | 1017 (22.8) | 433 (23.3) | 111 (20.0) |  |
|  | 63,000+ | 2014 (45.2) | 809 (43.6) | 257 (46.4) |  |
| Residence | Metro | 3687 (82.7) | 1514 (81.6) | 475 (85.7) | 0.008 |
|  | Urban | 454 (10.2) | 230 (12.4) | 44 (7.9) |  |
|  | Rural | 318 (7.1) | 111 (6.0) | 35 (6.3) |  |
| AJCC clinical T stage | cT1 | 833 (18.7) | 454 (24.5) | 129 (23.3) | <0.001 |
|  | cT2 | 588 (13.2) | 295 (15.9) | 104 (18.8) |  |
|  | cT3 | 1677 (37.6) | 682 (36.8) | 202 (36.5) |  |
|  | cT4 | 266 (6.0) | 58 (3.1) | 27 (4.9) |  |
|  | cTx | 1095 (24.6) | 366 (19.7) | 92 (16.6) |  |
| AJCC clinical N stage | cN0 | 2348 (52.7) | 1032 (55.6) | 327 (59.0) | <0.001 |
|  | cN1 | 1100 (24.7) | 477 (25.7) | 149 (26.9) |  |
|  | cN2 | 415 (9.3) | 131 (7.1) | 50 (9.0) |  |
|  | cN3 | 118 (2.6) | 44 (2.4) | 6 (1.1) |  |
|  | cNx | 478 (10.7) | 171 (9.2) | 22 (4.0) |  |
| Neoadjuvant therapy | None | 2359 (52.9) | 1002 (54.0) | 258 (46.6) | <0.001 |
|  | NCRT | 986 (22.1) | 471 (25.4) | 165 (29.8) |  |
|  | NAC | 1114 (25.0) | 382 (20.6) | 131 (23.6) |  |
| Tumor grade | Well | 252 (5.7) | 127 (6.8) | 30 (5.4) | 0.067 |
|  | Moderate | 1521 (34.1) | 671 (36.2) | 209 (37.7) |  |
|  | Poor | 2293 (51.4) | 900 (48.5) | 258 (46.6) |  |
|  | Anaplastic | 393 (8.8) | 157 (8.5) | 57 (10.3) |  |
| AJCC pathologic T stage | pT0 | 292 (6.5) | 147 (7.9) | 63 (11.4) | <0.001 |
|  | pT1 | 1218 (27.3) | 650 (35.0) | 203 (36.6) |  |
|  | pT2 | 670 (15.0) | 282 (15.2) | 62 (11.2) |  |
|  | pT3 | 1455 (32.6) | 590 (31.8) | 154 (27.8) |  |
|  | pT4 | 669 (15.0) | 147 (7.9) | 56 (10.1) |  |
|  | pTx | 155 (3.5) | 39 (2.1) | 16 (2.9) |  |
| AJCC pathologic N stage | pN0 | 2304 (54.8) | 1085 (60.6) | 341 (64.1) | <0.001 |
|  | pN1 | 835 (19.9) | 338 (18.9) | 84 (15.8) |  |
|  | pN2 | 556 (13.2) | 206 (11.5) | 64 (12.0) |  |
|  | pN3 | 330 (7.9) | 104 (5.8) | 29 (5.5) |  |
|  | pNx | 176 (4.2) | 56 (3.1) | 14 (2.6) |  |
| Lymphovascular invasion | Absent | 2107 (47.3) | 1002 (54.0) | 309 (55.8) | <0.001 |
|  | Present | 1455 (32.6) | 566 (30.5) | 142 (25.6) |  |
|  | Unknown | 897 (20.1) | 287 (15.5) | 103 (18.6) |  |
| 30-Day mortality | No | 4373 (98.1) | 1833 (98.8) | 542 (97.8) | 0.090 |
|  | Yes | 86 (1.9) | 22 (1.2) | 12 (2.2) |  |

CDCC,; AJCC, American Joint Committee on Cancer; NCRT, n**eoadjuvant chemoradiotherapy**; NAC, n**eoadjuvant chemotherapy**

**TABLE S12** Logistic regression of factors associated with textbook outcome for patients undergoing gastrectomy for gastric cancer in high-volume centers

|  |  | OR (univariable) *p* Value | OR (multivariable) *p* Value |
| --- | --- | --- | --- |
| Facility type | Integrated | — | — |
|  | Academic | 1.26 (1.03–1.55) 0.024 | 1.36 (1.07–1.74) 0.013 |
| Facility location | Northeast | — | — |
|  | Midwest | 0.60 (0.52–0.68) <0.001 | 0.61 (0.52–0.71) <0.001 |
|  | South | 0.48 (0.42–0.54) <0.001 | 0.51 (0.44–0.58) <0.001 |
|  | West | 0.83 (0.71–0.98) 0.023 | 0.85 (0.72–1.02) 0.078 |
| Hospital distance (miles) | <12.5 | — | — |
|  | 12.5–49.9 | 0.92 (0.82–1.03) 0.149 | 1.04 (0.91–1.18) 0.593 |
|  | ≥50 | 0.80 (0.71–0.90) <0.001 | 1.01 (0.86–1.18) 0.915 |
| Year of diagnosis | 2010–2011 | — | — |
|  | 2012–2013 | 1.18 (1.04–1.35) 0.012 | 1.19 (1.04–1.36) 0.014 |
|  | 2014–2015 | 1.32 (1.12–1.56) 0.001 | 1.29 (1.09–1.53) 0.003 |
|  | 2016–2017 | 1.61 (1.42–1.82) <0.001 | 1.54 (1.36–1.76) <0.001 |
| Age at diagnosis (years) | 18–35 | — | — |
|  | 36–50 | 1.20 (0.74–1.91) 0.455 | 1.06 (0.61–1.83) 0.828 |
|  | 51–65 | 1.14 (0.72–1.79) 0.565 | 1.04 (0.60–1.77) 0.895 |
|  | 66–80 | 1.00 (0.64–1.57) 0.991 | 1.02 (0.59–1.76) 0.947 |
|  | 80+ | 0.78 (0.49–1.25) 0.309 | 0.81 (0.46–1.42) 0.454 |
| Sex | Male | — | — |
|  | Female | 1.13 (1.02–1.26) 0.021 | 1.12 (1.01–1.26) 0.038 |
| Race | White | — | — |
|  | Other | 1.15 (1.04–1.28) 0.009 | 1.14 (1.01–1.29) 0.037 |
| CDCC score | 0 | — | — |
|  | 1–2 | 1.01 (0.91–1.12) 0.863 | 1.02 (0.91–1.13) 0.793 |
|  | 2+ | 0.78 (0.59–1.03) 0.076 | 0.76 (0.57–1.01) 0.061 |
| Insurance status | Medicare | — | — |
|  | Medicaid | 1.32 (1.09–1.59) 0.004 | 1.08 (0.87–1.35) 0.475 |
|  | Private | 1.34 (1.20–1.48) <0.001 | 1.31 (1.13–1.51) <0.001 |
|  | Not Insured / Other | 0.63 (0.49–0.82) 0.001 | 0.79 (0.60–1.04) 0.091 |
| Education level (%) | >21 | — | — |
|  | 13–20.9 | 0.83 (0.72–0.95) 0.007 | 0.93 (0.80–1.08) 0.337 |
|  | 7–12.9 | 0.87 (0.77–0.99) 0.037 | 0.91 (0.79–1.06) 0.238 |
|  | <7 | 0.84 (0.73–0.96) 0.011 | 0.86 (0.73–1.02) 0.089) |
| Medical income ($) | ≤47,999 | — | — |
|  | 48,000–62,999 | 1.00 (0.88–1.14) 0.946 | 1.02 (0.89–1.18) 0.763 |
|  | 63,000+ | 1.12 (1.01–1.25) 0.039 | 1.05 (0.91–1.21) 0.489 |
| Residence | Metro | — | — |
|  | Urban | 0.89 (0.76–1.03) 0.122 | 1.04 (0.87–1.25) 0.662 |
|  | Rural | 1.29 (1.06–1.57) 0.010 | 1.36 (1.10–1.68) 0.004 |
| AJCC clinical T stage | cT1 | — | — |
|  | cT2 | 1.34 (1.13–1.58) 0.001 | 1.21 (1.02–1.45) 0.031 |
|  | cT3 | 1.24 (1.09–1.41) 0.001 | 1.12 (0.95–1.32) 0.193 |
|  | cT4 | 1.11 (0.88–1.41) 0.380 | 0.91 (0.70–1.18) 0.489 |
|  | cTx | 1.04 (0.90–1.21) 0.560 | 1.04 (0.87–1.23) 0.686 |
| AJCC clinical N stage | cN0 | — | — |
|  | cN1 | 1.19 (1.06–1.34) 0.003 | 1.16 (1.01–1.33) 0.033 |
|  | cN2 | 1.14 (0.96–1.36) 0.144 | 1.04 (0.86–1.27) 0.680 |
|  | cN3 | 0.98 (0.72–1.34) 0.897 | 1.01 (0.72–1.41) 0.970 |
|  | cNx | 0.80 (0.68–0.94) 0.007 | 0.90 (0.74–1.09) 0.276 |
| Neoadjuvant therapy | None | — | — |
|  | NCRT | 1.00 (0.89–1.12) 0.979 | 0.91 (0.78–1.07) 0.249 |
|  | NAC | 1.49 (1.32–1.68) <0.001 | 1.29 (1.12–1.49) <0.001 |
| Surgical approach | Open | — | — |
|  | Laparoscopic | 1.37 (1.23–1.53) <0.001 | 1.22 (1.09–1.37) 0.001 |
|  | Robotic | 1.66 (1.38–2.00) <0.001 | 1.45 (1.19–1.76) <0.001 |

OR, odds ratio; CDCC,; AJCC, American Joint Committee on Cancer; NCRT, n**eoadjuvant chemoradiotherapy**; NAC, n**eoadjuvant chemotherapy**

**TABLE S13** Cox regression of factors associated with long-term survival of patients undergoing gastrectomy for gastric cancer in high-volume (Quintile 5) centers

|  |  | HR (univariable) *p* Value | HR (multivariable) *p* Value |
| --- | --- | --- | --- |
| Facility type | Integrated | — | — |
|  | Academic | 1.05 (0.89–1.25) 0.549 | 1.04 (0.85–1.28) 0.685 |
| Facility location | Northeast | — | — |
|  | Midwest | 1.42 (1.27–1.58) <0.001 | 1.14 (1.01–1.28) 0.027 |
|  | South | 1.42 (1.29–1.56) <0.001 | 1.17 (1.06–1.30) 0.002 |
|  | West | 1.19 (1.05–1.35) 0.007 | 1.24 (1.08–1.42) 0.002 |
| Hospital distance (miles) | <12.5 | — | — |
|  | 12.5–49.9 | 1.35 (1.23–1.48) <0.001 | 1.19 (1.07–1.32) 0.001 |
|  | ≥50 | 1.63 (1.49–1.79) <0.001 | 1.23 (1.09–1.39) 0.001 |
| Year of diagnosis | 2010–2011 | — | — |
|  | 2012–2013 | 0.93 (0.85–1.03) 0.159 | 0.97 (0.88–1.06) 0.487 |
|  | 2014–2015 | 0.85 (0.75–0.97) 0.015 | 0.96 (0.85–1.10) 0.577 |
|  | 2016–2017 | 0.84 (0.75–0.93) 0.002 | 0.91 (0.81–1.02) 0.122 |
| Age at diagnosis (years) | 18–35 | — | — |
|  | 36–50 | 0.98 (0.65–1.48) 0.911 | 1.16 (0.72–1.84) 0.544 |
|  | 51–65 | 0.99 (0.67–1.48) 0.973 | 1.20 (0.76–1.91) 0.437 |
|  | 66–80 | 1.21 (0.82–1.80) 0.340 | 1.38 (0.86–2.21) 0.184 |
|  | 80+ | 1.92 (1.28–2.89) 0.002 | 2.15 (1.33–3.48) 0.002 |
| Sex | Male | — | — |
|  | Female | 0.89 (0.82–0.97) 0.009 | 0.94 (0.86–1.03) 0.178 |
| Race | White | — | — |
|  | Other | 0.74 (0.67–0.81) <0.001 | 0.83 (0.75–0.92) <0.001 |
| CDCC score | 0 | — | — |
|  | 1–2 | 1.09 (1.00–1.18) 0.051 | 1.07 (0.98–1.17) 0.127 |
|  | 2+ | 1.49 (1.21–1.85) <0.001 | 1.54 (1.24–1.91) <0.001 |
| Insurance status | Medicare | — | — |
|  | Medicaid | 0.69 (0.58–0.82) <0.001 | 0.85 (0.70–1.02) 0.083 |
|  | Private | 0.72 (0.66–0.78) <0.001 | 0.83 (0.74–0.93) 0.001 |
|  | Not Insured / Other | 0.83 (0.68–1.03) 0.088 | 0.85 (0.68–1.06) 0.155 |
| Education level (%) | >21 | — | — |
|  | 13–20.9 | 1.61 (1.44–1.80) <0.001 | 1.38 (1.23–1.56) <0.001 |
|  | 7–12.9 | 1.55 (1.39–1.72) <0.001 | 1.50 (1.33–1.69) <0.001 |
|  | <7 | 1.31 (1.16–1.47) <0.001 | 1.46 (1.27–1.68) <0.001 |
| Medical income ($) | ≤47,999 | — | — |
|  | 48,000–62,999 | 0.90 (0.81–0.99) 0.033 | 0.80 (0.72–0.90) <0.001 |
|  | 63,000+ | 0.67 (0.61–0.73) <0.001 | 0.66 (0.59–0.74) <0.001 |
| Residence | Metro | — | — |
|  | Urban | 1.55 (1.39–1.72) <0.001 | 1.17 (1.03–1.33) 0.014 |
|  | Rural | 0.56 (0.46–0.68) <0.001 | 0.50 (0.41–0.61) <0.001 |
| AJCC clinical T stage | cT1 | — | — |
|  | cT2 | 1.72 (1.48–1.99) <0.001 | 1.64 (1.40–1.91) <0.001 |
|  | cT3 | 2.29 (2.03–2.58) <0.001 | 2.00 (1.73–2.31) <0.001 |
|  | cT4 | 2.63 (2.19–3.15) <0.001 | 2.46 (2.02–3.00) <0.001 |
|  | cTx | 2.05 (1.80–2.34) <0.001 | 1.95 (1.68–2.26) <0.001 |
| AJCC clinical N stage | cN0 | — | — |
|  | cN1 | 1.51 (1.38–1.65) <0.001 | 1.27 (1.14–1.41) <0.001 |
|  | cN2 | 1.77 (1.55–2.01) <0.001 | 1.59 (1.38–1.84) <0.001 |
|  | cN3 | 2.39 (1.95–2.93) <0.001 | 1.93 (1.55–2.39) <0.001 |
|  | cNx | 1.52 (1.34–1.72) <0.001 | 1.24 (1.07–1.45) 0.005 |
| Neoadjuvant therapy | None | — | — |
|  | NCRT | 1.41 (1.29–1.55) <0.001 | 1.01 (0.89–1.14) 0.884 |
|  | NAC | 1.15 (1.04–1.26) 0.005 | 0.96 (0.86–1.08) 0.495 |
| Textbook outcome | No | — | — |
|  | Yes | 0.56 (0.52–0.61) <0.001 | 0.58 (0.54–0.63) <0.001 |
| Surgical approach | Open | — | — |
|  | Laparoscopic | 0.83 (0.76–0.91) <0.001 | 0.95 (0.86–1.04) 0.255 |
|  | Robotic | 0.79 (0.67–0.93) 0.004 | 0.82 (0.73–0.91) 0.003 |

HR, hazard ratio; CDCC,; AJCC, American Joint Committee on Cancer; NCRT, n**eoadjuvant chemoradiotherapy**; NAC, n**eoadjuvant chemotherapy**
